# Association between the Changes in Trimethylamine N-Oxide-Related Metabolites and Prognosis of Patients with Acute Myocardial Infarction: A Prospective Study

**DOI:** 10.3390/jcdd9110380

**Published:** 2022-11-04

**Authors:** Nan Li, Ying Wang, Jinying Zhou, Runzhen Chen, Jiannan Li, Xiaoxiao Zhao, Peng Zhou, Chen Liu, Yi Chen, Li Song, Hanjun Zhao, Hongbing Yan, Shaodi Yan

**Affiliations:** 1 Department of Cardiology, Fuwai Hospital, National Center for Cardiovascular Diseases, Peking Union Medical College & Chinese Academy of Medical Sciences, Beijing 100037, China; 2 Department of Cardiology, Fuwai Hospital, Chinese Academy of Medical Sciences, Shenzhen 518057, China

**Keywords:** acute myocardial infarction, gut microbiome-driven metabolites, changes in trimethylamine N-oxide, changes in choline

## Abstract

This study aimed to investigate the association between changes in levels of trimethylamine N-oxide (TMAO) and its precursors and the prognosis of patients with acute myocardial infarction (AMI). Patients diagnosed with AMI were prospectively enrolled at Fuwai Hospital between March 2017 and January 2020. TMAO, betaine, choline, and L-carnitine were measured in 1203 patients at their initial admission and 509 patients at their follow-up of one month. Major adverse cardiovascular events (MACE), a composite of all-cause death, recurrence of MI, rehospitalization caused by HF, ischemic stroke, and any revascularization, were followed up. A decision tree by TMAO levels implicated that compared to those with low levels at admission, patients with high TMAO levels at both time points showed an increased risk of MACE (adjusted hazard ratio (HR) 1.59, 95% confidence interval (CI): 1.03–2.46; *p* = 0.034), while patients with high TMAO levels at admission and low levels at follow-up exhibited a similar MACE risk (adjusted HR 1.20, 95% CI: 0.69–2.06; *p* = 0.520). Patients with high choline levels at admission and follow-up showed an elevated MACE risk compared to those with low levels at both time points (HR 1.55, 95% CI: 1.03–2.34; *p* = 0.034). Repeated assessment of TMAO and choline levels helps to identify the dynamic risk of cardiovascular events.

## 1. Introduction

Increasing studies have suggested the associations between metabolites of intestinal microbiota and cardiovascular diseases [1,2]. Trimethylamine N-oxide (TMAO), a classic component of the metabolic pathway, has been extensively investigated and regarded as a biomarker for adverse cardiovascular events [3,4]. Studies have shown the association between TMAO levels and worse outcomes in cardiovascular diseases [4,5,6,7,8]. TMAO could also improve the identification of coronary culprit plaque rupture in acute myocardial infarction (AMI) [9]. Moreover, several studies have already focused on the impact of serial TMAO levels on cardiovascular diseases. Heianza et al. implied that long-term elevation in TMAO levels was associated with increased coronary heart disease (CHD) risk in healthy women, and repeated measurement of TMAO over ten years could identify high CHD risk [10]. Another study reported that elevated TMAO levels at enrolment and nine months after treatment were correlated with a higher risk of adverse events in patients with chronic heart failure (HF) [11]. However, little is known about changes in TMAO levels and prognosis in the population with AMI. The response of TMAO levels to pharmacological treatment in these patients is also unknown.

TMAO is generated from the oxidation of trimethylamine (TMA), which comes from betaine, choline, and L-carnitine [12]. A large body of studies has investigated the associations of betaine, choline, and L-carnitine with cardiometabolic risk and mortality among different populations. A study that included patients with stable coronary artery disease found that higher plasma choline and betaine levels were risk factors for major adverse cardiovascular events (MACE) in a concomitant increase in TMAO levels [13]. Moreover, the Jackson Heart Study results implied that high dietary betaine intake was correlated to the high CHD incidence, whereas higher dietary choline intake was not [14]. As for L-carnitine, Koeth et al. conducted a study that enrolled patients undergoing cardiac evaluation and reported that higher L-carnitine levels indicated an elevated risk of cardiovascular disease and incidence of MACE only with concurrently high TMAO levels [15]. However, a systematic review summarized that the results did not support a positive association between dietary choline or betaine and the incidence of cardiovascular diseases in healthy people [16]. Given the inconsistent results, the impact of betaine, choline, and L-carnitine should be further investigated. 

Therefore, this study focused on patients with AMI and investigated the association of TMAO, betaine, choline, and L-carnitine levels with prognosis and further explored the association of changes in these levels during follow-up with prognosis. 

## 2. Methods

### 2.1. Study Population

Between March 2017 and January 2020, we prospectively recruited patients admitted to the emergency department of Fuwai Hospital with a diagnosis of AMI. AMI is defined by the Fourth Universal Definition of Myocardial Infarction and guidelines, including elevated troponin I level and clinical evidence of ischemia such as sustained chest pain, new ST-segment changes, and new regional wall motion abnormality [17,18,19]. A flowchart of the patient selection process is presented in Supplemental Figure S1. Patients who were missing gut metabolite levels or follow-up records were excluded. Information on demographics, physical examinations, medical histories, laboratory results, echocardiography data, and medication at discharge was collected through the hospital information system. The Ethics Committee of Fuwai Hospital approved the current study, which conformed to the Declaration of Helsinki. All patients enrolled signed informed consent.

### 2.2. Outcomes and Follow-Up

The primary outcome of this study was MACE, including all-cause death, recurrence of MI, rehospitalization caused by HF, stroke, and any revascularization. HF was identified following guidelines and statements up to date, based on typical symptoms and signs, laboratory tests, echocardiogram, and X-ray findings [20,21]. Outpatient visits and telephone interviews were used to collect outcome data and conducted with patients at 1, 6, and 12 months after discharge and then annually. The Institutional Review Board of Fuwai Hospital approved the protocol for follow-up.

### 2.3. Sample Collection and Tests

Blood samples were taken from the radial or femoral artery before heparinization and percutaneous coronary intervention (PCI) at admission (V1) and the cubital vein approximately one month later when patients came to the clinic (V2). The samples were maintained with tubes containing ethylenediaminetetraacetic at 4 °C, centrifuged within 3 h, the supernatant was separated and stored at −80 °C until subsequent analysis. As described in previous studies, plasma levels of TMAO and its precursors were measured by stable isotope dilution high-performance liquid chromatography with online electrospray ionization tandem mass spectrometry using an API 3200 triple quadrupole mass spectrometer (AB SCIEX, Framingham, MA, USA) with a d9-TMAO, d9-betaine, d9-choline, and d3-carnitine internal standard [22,23]. The other blood parameters were routinely measured in the hospital’s central laboratory. 

### 2.4. Statistical Analyses

Baseline characteristics were displayed as mean ± standard deviation or median with interquartile range (IQR) for continuous variables and number (percentage) for categorical variables. Selecting the appropriate analysis method according to the number of groups and variable type compared the differences among groups. For patients who completed the second visit, the changes of continuous variables from V1 to V2 were compared by the Wilcoxon matched-pairs signed-rank test. 

To investigate the association between TMAO and its precursors and cardiovascular risk in AMI, we grouped the patients based on plasma median and teritle levels of TMAO and its precursors, respectively. An analysis of the Kaplan–Meier curve and log-rank test was used to determine the event-free survival rates of the groups. Univariable and multivariable Cox proportional hazards regression analyses were used to compute the adjusted hazard ratios (HRs) and 95% confidence intervals (CIs). The variables with *p* < 0.1 in the univariable models were further analyzed in the multivariable Cox regression. Furthermore, the adjusted factors would add TMAO levels for the analysis of the precursors. Possible nonlinear relationships were accessed using restricted cubic spline (RCS) regression with TMAO as a continuous variable with four knots, which also adjusted for the confounders mentioned above.

To explore the relationship between changes in TMAO levels and subsequent MACE after V2, we classified patients with available data for both time points based on the respective median levels of TMAO at each time point. Then we created four groups, consisting of L/L group (low V1 and low V2), L/H group (low V1 and high V2), H/L group (high V1 and low V2), and H/H group (high V1 and high V2). Cox regression analysis examined the associations between each group and outcomes. The HRs of MACEs among groups were displayed in forest plots. A decision tree analysis was performed by the 𝜒2 automatic interaction detection to generate groups of different MACE risk and the association of resultant groups with MACE was confirmed by multivariable Cox regression and Kaplan–Meier survival curves. Furthermore, betaine, choline, and L-carnitine levels were analyzed similarly.

The SPSS software (version 26.0; IBM Corp., Armonk, NY, USA) and R (http://www.r-project.org/, (accessed on 1 March 2022) statistical packages were used to analyze the data. *p* < 0.05 was set out as statistically significant.

## 3. Results

### 3.1. Characteristics of Included Patients

Between March 2017 and January 2020, 1203 patients admitted to the emergency department of Fuwai Hospital and diagnosed with AMI were analyzed in this study. The baseline characteristics of the included patients are listed in Table 1. The median age of all patients was 61 (IQR 53–69) years, and 963 (80.0%) patients were male. The median plasma levels of TMAO, betaine, choline, and L-carnitine in all patients were 6.6 (IQR 4.0–11.6) µmol/L, 1.9 (IQR 1.5–2.4) µmol/L, 1.2 (IQR 1.0–1.5) µmol/L, and 50.6 (IQR 42.5–59.6) µmol/L, respectively. The patient characteristics grouped by the respective median and tertile levels of TMAO, betaine, choline, and L-carnitine are presented in Supplemental Tables S1–S4. Patients with the third tertile levels of TMAO and choline were older and presented with a higher rate of Killip II-IV, previous stroke, diabetes mellitus, and chronic kidney disease (CKD), and a higher frequency of MACE during follow-up. Moreover, the levels of betaine, choline and L-carnitine showed an increasing trend according to the tertile levels of TMAO. 

The follow-up levels of TMAO, betaine, choline, and L-carnitine were available in 509 (42.3%) patients. These patients showed an improvement in clinical variables (Table 1) after one month of treatment. The levels of low-density lipoprotein cholesterol (*p* < 0.001) and high-sensitivity C-reactive protein (*p* < 0.001) reduced significantly, while TMAO and its precursors all exhibited an increasing trend (*p* < 0.001). The Supplemental Table S5 summarizes characteristic differences between patients with and without TMAO measurements at V2. Patients without TMAO measurements at V2 presented with a higher prevalence of previous CKD (*p* = 0.001) and Killip II-IV (*p* < 0.001), as well as a higher incidence of MACE (*p* = 0.011) and all-cause death (*p* = 0.011).

### 3.2. Association between Plasma Levels of TMAO, Betaine, Choline, and L-Carnitine at Baseline and Adverse Outcomes

**Figure 1:**
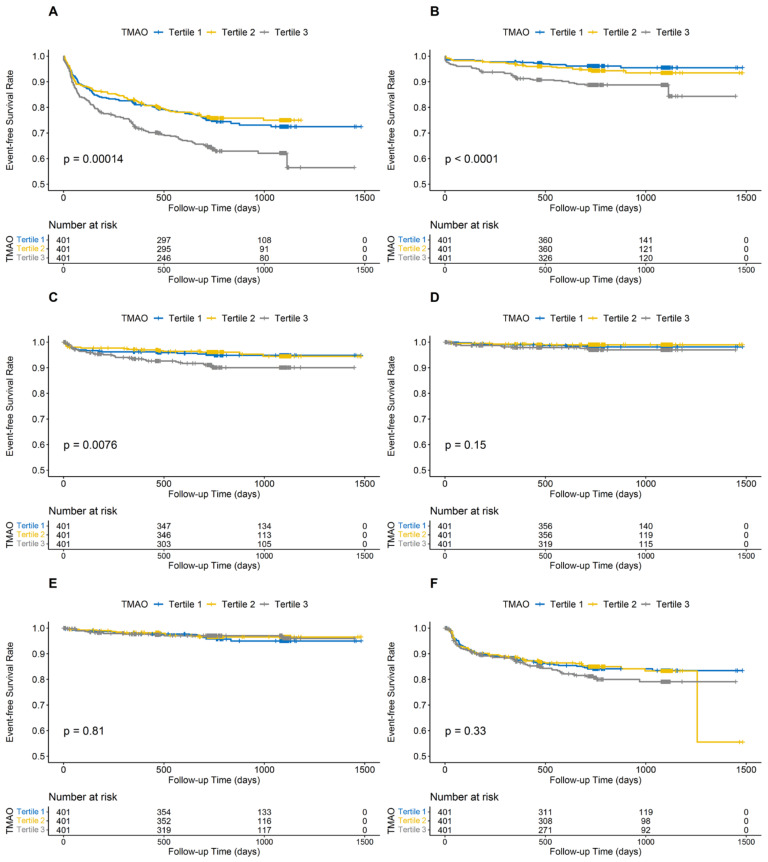
Kaplan—curve for cumulative event-free survival in groups stratified by TMAO tertile levels at enrollment. (**A**) major adverse cardiovascular event, (**B**) all-cause death, (**C**) myocardial infarction, (**D**) rehospitalization caused by heart failure; (**E**) stroke; (**F**) revascularization. TMAO, trimethylamine-N-oxide.

A total of 343 (28.5%) patients experienced MACE during a median time of 739 days follow-up. The Kaplan–Meier analysis of groups classified by the tertile TMAO levels in Figure 1 revealed that MACE risk in tertile 3 increased significantly (*p* < 0.001), as well as the risk for all-cause death (*p* < 0.001) and recurrent MI (*p* = 0.008). As shown in Table 2, compared with patients in tertile 1 (<4.76 µmol/L), those in tertile 3 had a higher MACE risk (>9.38 µmol/L, adjusted HR: 1.35, 95% CI: 1.02–1.78; *p* = 0.033), as well as risks of all-cause death and recurrent MI (HR: 2.06, 95% CI: 1.01–4.19; *p* = 0.047 and HR:1.95, 95% CI: 1.08–3.53; *p* = 0.027, respectively). As a continuous variable, the RCS regression analysis curved an S-shaped relationship between TMAO levels and HR for MACE and all-cause death (*p* for nonlinearity = 0.045 and 0.004, respectively) after adjusting for the confounding factors (Supplemental Figure S2). 

For patients grouped by choline tertile levels, the Kaplan–Meier analysis in Supplemental Figure S3 showed that there was no statistical difference in MACE risk, whereas patients in tertile 3 had higher risks of all-cause death (*p* < 0.001) and rehospitalization caused by HF (*p* = 0.024). Moreover, the Kaplan–Meier analysis of betaine tertile levels (Supplemental Figure S4) revealed that patients in tertile 3 had an increased risk of rehospitalization caused by HF (*p* = 0.026), while for L-carnitine (Supplemental Figure S5), there were no statistical differences in any endpoints. The details of Cox regression of relationships between all endpoints and choline, betaine, and L-carnitine levels are shown in Supplemental Tables S6–S8. After adjusting the confounders, the HR for rehospitalization caused by HF was higher in patients above the median choline levels (HR 2.90, 95% CI: 1.02–8.25; *p =* 0.045). Apart from this, the other HRs of all endpoints were not statistically different. 

### 3.3. Association between Changes in Levels of TMAO, Betaine, Choline, and L-Carnitine and Adverse Outcomes

**Figure 2:**
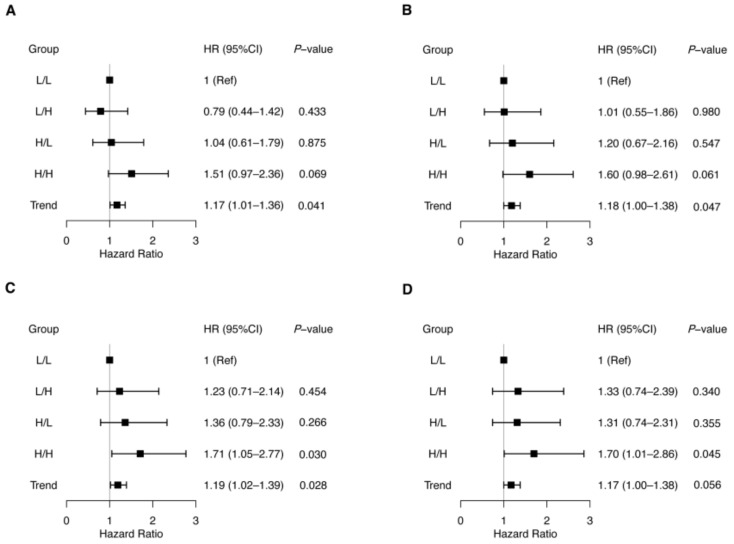
Forest plot of hazard ratios for major adverse cardiovascular events in groups according to trimethylamine N-oxide (TMAO) (**A**,**B**) and choline (**C**,**D**) levels at enrollment (V1) and follow-up visit (V2). Patients with available TMAO and choline levels of V1 and V2 were divided into four groups according to the median levels of each visit (TMAO: 6.7 µmol/L and 12.7 µmol/L, choline: 1.2 µmol/L and 1.7 µmol/L for V1 and V2, respectively). L/L, low V1 and low V2; L/H, low V1 and high V2; H/L, high V1 and low V2; H/H, high V1 and high V2. Cox proportional hazards regression was used to compare the risk of major adverse cardiovascular events among the four groups of patients using L/L as the reference on each occasion [(**A**,**C**) unadjusted, (**B**) adjusted with age, hypertension, diabetes, peripheral artery disease, chronic kidney disease, and previous history of stroke and MI, Killip II-IV, the Global Registry of Acute Coronary Events risk score, multiple vessels disease, percutaneous coronary intervention, and the peak value of cardiac troponin I and N-terminal pro-B-type natriuretic peptide during hospitalization, as well as estimated glomerular filtration rate and left ventricular ejection fraction at V2; (**D**) adjusted with these factors and TMAO levels at V2].

To investigate the associations between serial levels of TMAO following treatment and subsequent adverse events, we divided the patients with available TMAO levels of V1 and V2 into four groups according to the median levels of each visit (V1: 6.7 µmol/L; V2: 12.7 µmol/L). These groups were comprised of L/L group (*n* = 155), L/H group (*n* = 99), H/L group (*n* = 99), and H/H group (*n* = 156). The forest plot in Figure 2A showed that compared to the L/L group, no statistical differences were observed in each group (L/H group: HR 0.79, 95% CI: 0.44–1.42, *p* = 0.433; H/L group: HR 1.04, 95% CI: 0.61–1.79, *p* = 0.875; H/H group: HR 1.51, 95% CI: 0.97–2.36, *p* = 0.069), while the trend showed significantly increasing (adjusted HR 1.18, 95% CI: 1.00–1.38, *p* = 0.047). Moreover, there was no statistical significance in TMAO levels and individual endpoints (Table 3).

We also grouped the 509 patients based on the median betaine, choline, and L-carnitine levels. As for choline, the median levels of V1 and V2 were 1.2 µmol/L and 1.7 µmol/L, respectively. These groups were comprised of L/L group (*n* = 147), L/H group (*n* = 107), H/L group (*n* = 107), and H/H group (*n* = 148). After adjusting for confounders and TMAO levels at V2, no statistical significance of MACE risk was seen in the L/H and H/L groups compared to the L/L group (HR 1.33, 95% CI: 0.74–2.39; *p* = 0.340 and HR 1.31, 95% CI: 0.74–2.31; *p* = 0.355, respectively; Figure 2D), while the H/H group showed an increased risk of MACE (adjusted HR 1.70, 95% CI: 1.01–2.86; *p* = 0.045; Figure 2D). However, there was no statistical significance in choline levels and individual endpoints (Supplemental Table S9). As for betaine and L-carnitine levels, no positive associations were obtained in composite and individual endpoints, and the details are shown in Supplemental Tables S10 and S11.

### 3.4. Decision Tree Analysis

**Figure 3:**
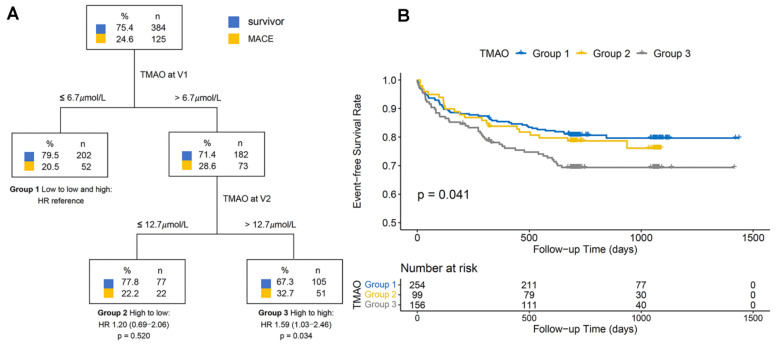
Decision tree of risk stratification for major adverse cardiovascular events (MACE) using combined measurements at enrollment (V1) and follow-up visit (V2) for trimethylamine N-oxide (TMAO) (**A**). Kaplan–Meier curve for cumulative MACE-free survival in groups generated by decision tree (**B**). Decision tree using plasma TMAO level at V1 as the initial classifier, followed by plasma TMAO level at V2 enables effective selection of low- and high-risk groups of patients and increased cumulative event risk in Group 3 compared to Group 1. The number of events is shown below. Data are presented as adjusted hazard ratio (HR) and 95% confidence interval (CI). The adjusted factors included age, hypertension, diabetes, peripheral artery disease, chronic kidney disease, previous history of stroke and MI, Killip II-IV, the Global Registry of Acute Coronary Events risk score, multiple vessels disease, percutaneous coronary intervention, and the peak value of cardiac troponin I and N-terminal pro-B-type natriuretic peptide during hospitalization, as well as estimated glomerular filtration rate and left ventricular ejection fraction at V2.

The decision tree analysis was performed to explore the TMAO levels at baseline and follow-up as biomarkers for risk stratification of MACE after V2 (Figure 3A). Classifying the patients according to the median TMAO levels at V1 and V2 created three risk groups and verified by the Kaplan–Meier analysis (Figure 3B) and multivariable Cox regression analysis (Table 3). The results revealed that patients who presented with high TMAO levels at V1 and low levels at V2 (Group 2, HR 1.20, 95% CI: 0.69–2.06; *p* = 0.520) had a similar risk for MACE to those with low TMAO levels at V1 (Group 1), whereas those who presented with high TMAO levels at V1 and V2 showed an increased MACE risk (Group 3, adjusted HR 1.59, 95% CI: 1.03–2.46; *p* = 0.034). 

However, the decision tree analysis could not be done based on the choline, betaine, and L-carnitine levels. In addition, we divided patients into three groups simply according to median choline levels at V1 and V2 without applying decision tree approach, namely Group 1 (patients with low choline levels at V1), Group 2 (high choline levels at V1 and subsequently low levels at V2) and Group 3 (high choline levels at V1 and V2). The Cox regression analysis (Supplemental Table S9) suggests that patients in Group 3 showed an increased risk of MACE (HR 1.55, 95% CI: 1.03–2.34; *p* = 0.034) compared to those in Group 1, whereas the difference diminished after adjusting for the confounders and TMAO levels of V2. Moreover, the Kaplan–Meier analysis (Supplemental Figure S6) shows no significant differences in MACE risk among groups (*p* = 0.100). 

## 4. Discussion

This study set out to investigate the association of TMAO and its precursor (betaine, choline, and L-carnitine) levels with prognosis and the relationship between changes in these levels during follow-up and prognosis in a population with AMI. Firstly, the analysis in this study confirmed that TMAO levels at baseline were correlated to an increased risk of MACE, with an S-shaped relationship. Secondly, only patients with high TMAO levels at V1 and V2 showed an increase in MACE risk, while the risk of MACE in patients with high TMAO levels at V1 and low levels at V2 did not increase significantly. A similar condition was seen in choline levels. Changes in TMAO and choline levels during follow-up could indicate dynamic MACE risk. 

### 4.1. Association between Levels of TMAO and Its Precursors and Prognosis

Substantial clinical studies have established that TMAO was strongly associated with poor outcomes in cardiovascular diseases [3,24]. Animal studies also have confirmed the causality between TMAO and atherosclerosis [25] and demonstrated that TMAO could accelerate atherosclerosis, thrombosis, cardiorenal fibrosis, and vascular inflammation through different pathways, resulting in the progression of CHD [26]. This study found that higher TMAO levels at baseline were independently correlated to an elevated MACE risk, as the same with the previous studies focused on acute coronary syndrome or stable coronary syndrome [7,27,28]. Thus, these findings implied that TMAO was a vital risk factor for cardiovascular events and measuring TMAO levels could help identify MACE risk in CHD patients. 

Choline is critical in the process of lipid metabolism, cell membrane structure, and cholinergic neurotransmission [29]. As a dietary precursor of TMAO, choline is a risk factor for cardiovascular diseases and death. Betaine, a metabolite of choline, is also regarded as a potential risk factor for cardiovascular diseases. Wang et al. demonstrated that higher betaine (HR 1.33, 95% CI: 1.03–1.73; *p* < 0.05) and choline (HR 1.34, 95% CI: 1.03–1.74; *p* < 0.05) levels could each predict an elevated risk for cardiovascular events (including death, MI and stroke) after adjusted traditional cardiovascular risk factors in patients with stable coronary disease, while the addition of TMAO completely attenuated these association [13]. Yang et al. reported that plasma betaine levels were correlated to the disease severity among patients with pulmonary hypertension [30]. However, not all clinical studies of choline and betaine showed consistent results. The present study showed that high betaine and choline levels at baseline were not associated with an increased risk of MACE. Similarly, Trøseid et al. demonstrated that either choline or betaine was not associated with an increased risk of heart transplantation in patients with chronic HF [31]. As for another precursor of TMAO, L-carnitine plays a crucial role in lipid metabolism [32]. Koeth et al. implied a positive association of L-carnitine with poor prognosis among patients undergoing cardiac evaluation, while the association diminished after adjusting for TMAO levels [15]. However, the present study did not observe associations between L-carnitine levels at baseline and the risk of MACE. Regarding the inconsistent results of TMAO’s precursors and prognosis, a possible explanation may be that the study population and endpoints are different. Furthermore, the different approaches to measurement in studies may also influence the results. Although current studies would not draw a clear conclusion about this matter, these findings still remind us of the potential roles of choline, betaine, and L-carnitine in the risk management of cardiovascular diseases. More studies in this field are needed and may clarify the association between betaine, choline and L-carnitine and prognosis.

### 4.2. Association between Serial Levels of TMAO and Its Precursors and Prognosis

There were a few studies focused on serial levels of TMAO. Suzuki et al. conducted a study [11] of serial TMAO levels during a nine-month follow-up in patients with chronic HF. They demonstrated that patients with higher TMAO levels at both time points showed an elevated risk of all-cause death at 2 years (HR 2.10, 95% CI: 1.44–3.06; *p* < 0.001), whereas patients with higher TMAO levels at the first time and lower at the second time showed a similar risk of all-cause death at 2 years to those with lower TMAO levels at the first time. In addition, a study by Heianza et al. explored the association between ten-year changes in TMAO levels and CHD incidence in healthy women [10]. The results pointed out that long-term increases in TMAO levels could predict higher CHD risk (HR 1.79, 95% CI: 1.08–2.96; *p* = 0.023) compared to low TMAO levels at the first and second times [10]. Likewise, the present study found that TMAO levels at a follow-up of one month could help stratify the patients at high risk of MACE. The results indicated that patients with high TMAO levels at both time points had 1.59 times (95% CI: 1.03–2.46; *p* = 0.034) higher risk of MACE than those with low TMAO levels at V1, while the MACE risk of patients with high TMAO levels at V1 and low levels at V2 did not increase significantly compared to those with low TMAO levels at V1 (HR 1.20, 95% CI: 0.69–2.06; *p* = 0.520). However, the risk of MACE in the H/H group did not increase significantly compared to the L/L group; only the trend among the groups divided according to the respective median levels showed statistically significant (HR 1.17, 95% CI: 1.01–1.36; *p* for trend = 0.041). A possible explanation for this is that patients who did not complete the follow-up visit had a nearly two times higher mortality than those with follow-up data (8.6% vs. 4.7%, *p* = 0.011). In other words, the conditions of patients who completed the follow-up visit were relatively mild, implying a lower risk of MACE. Except for this, our earlier study observed that high TMAO levels were associated with more prevalence of culprit plaque rupture, which would result in high mortality [9]. This may partially explain the results of this study. In brief, these findings suggested that repeated assessment of TMAO levels during follow-up could predict changes in MACE risk. 

Meanwhile, this study further explored whether the changes in levels of betaine, choline and L-carnitine were associated with prognosis. The results revealed that only those with higher choline levels at both time points were correlated to an increased risk of MACE compared to patients with lower levels at both time points (adjusted HR 1.70, 95% CI: 1.01–2.86; *p* = 0.045). Although these results were not very encouraging, this information could still remind us that repeated measurements of choline levels may be beneficial for long-term risk assessment in patients with AMI. Moreover, it is worth noting that this finding still needs to be verified in a large sample.

### 4.3. Metabolism and Function of TMAO and Its Precursors and Clinical Implications 

TMA is generated in the gut from dietary betaine, choline, and other choline-containing compounds, and L-carnitine. These precursors are converted into TMA by various enzymes, such as betaine reductase, carnitine oxidoreductase, and choline TMA lyase [12]. Most TMA ingested or formed in the gut is rapidly absorbed into the portal circulation by passive diffusion and then oxidized to TMAO by hepatic flavin containing monooxygenases FMO3 and FMO1. Nearly 95% of TMA is oxidized and afterwards excreted in the urine in a 3:95 TMA: TMAO ratio within 24 h, only 4% is excreted in feces, and less than 1% is eliminated in the breath [33]. 

Numerous studies have investigated the mechanism of TMAO association with poor prognosis in cardiovascular diseases. Firstly, TMAO could suppress reverse cholesterol transport by modulating bile acid pool size and composition, accelerate foam cell formation by upregulating cluster of differentiation 36 and scavenger receptor A located on macrophages, and enhance uptake of oxidized low-density lipoprotein cholesterol [34,35]. Secondly, TMAO could activate inflammatory cascades via different pathways, such as mitogen-activated protein kinase signaling, nucleotide-binding oligomerization domain-like receptor family pyrin domain-containing 3 inflammasome, and nuclear factor-κB signaling pathway [36,37,38]. Furthermore, TMAO could enhance platelet reactivity through altering platelet calcium signaling and may diminish the anti-platelet effect [6,39]. These underlying mechanisms of TMAO might all contribute to the progression of cardiovascular diseases and imply that TMAO is a therapeutic target for cardiovascular diseases and deserves more attention and research. To date, some studies have found various methods to reduce TMAO levels. For example, ginkgolide B, an herbal component from Ginkgo biloba leaves, could inhibit the expression of flavin monooxygenase 3 (a hepatic enzyme in the transition from trimethylamine to TMAO) and then reduce TMAO levels [40]. Moreover, changing the intestinal microbiome’s composition also could reduce TMAO levels, including antibiotics, anti-diabetic treatment, and choline analogue [41,42,43]. However, the impact of reducing TMAO levels on the prognosis of cardiovascular disease remains unclear, and further studies are needed.

Except as a precursor of TMAO, choline is an essential nutrient for humans. Foods of animal origin, especially eggs and liver, are rich in choline; most foods we eat contain some amounts of choline or choline compounds. Choline has four primary metabolites: acetylcholine, betaine, phospholipids, and TMA [44]. Correspondingly, acetylcholine is the neurotransmitter and binds to receptors of the post-synaptic neuron in the central and peripheral nervous systems; phospholipids assure the structural integrity and signaling functions of cell membranes [44]. Betaine mainly comes from plant origins, such as spinach, beets and grains. It serves as an osmolyte and a methyl group donor and is involved in homocysteine methyltransferase reaction [29,45]. Moreover, phosphatidylcholine, synthesised from choline, is involved in very low-density lipoprotein production [44]. Studies also demonstrated that choline intake would lead to cholesterol and triglyceride transport from the liver to the vessels, causing cholesterol and triglyceride levels increased [46]. However, a study [47] reported that egg (a food rich in choline) intake could cause high-density lipoprotein cholesterol and choline concentrations to increase and no change in plasma low-density lipoprotein cholesterol or TMAO concentrations, which implied the complex association between choline and lipid metabolism. The specific interaction needs further investigation in the future.

L-carnitine, another precursor of TMAO, is found in animal products such as meat, fish, poultry, and milk. It could be transformed into betaine and γ-butyrobetaine [33]. First, L-carnitine plays a critical role in fat metabolism, transporting the activated long-chain fatty acids from the cytosol into the mitochondria and making them available for mitochondrial β-oxidation [48]. Second, carnitine may suppress the accumulation of lactic acid by reacting with acyl-coenzyme A to form acetyl-carnitine and coenzyme A, thereby enhancing high-intensity exercise performance [48]. Moreover, experimental data illustrated that carnitine administration could increase serum osteocalcin concentrations in animals, which suggested that carnitine might be helpful for the prevention and/or therapeutic treatment of osteoporosis and post-menopause syndrome [49]. Concerning the essential role of L-carnitine, enriching food types and maintaining normal L-carnitine levels is crucial.

## 5. Limitations 

There were some limitations of this study. Firstly, we only assessed the levels of TMAO and its precursors before PCI and one month after admission. These two-time points did not perfectly reflect the changes in TMAO, betaine, choline, and L-carnitine levels. In addition, we did not collect the diet of these patients during follow-up. Secondly, we enrolled patients admitted to the emergency department with a relatively higher mortality risk. Attention should be paid to the characteristics of patients included in this study when interpreting and generalizing the results and conclusions. Thirdly, to some degree, the small number of patients who completed both visits resulted in fewer adverse events. Therefore, larger sample prospective studies on serial levels of TMAO, betaine, choline, and L-carnitine in different populations would help us establish greater accuracy on this matter. 

## 6. Conclusions

Repeated assessment of TMAO and choline levels during follow-up could identify changes in MACE risk in patients with AMI. Serial increased levels of choline and TMAO at baseline and one-month follow-up each indicated an increased cardiovascular risk. 

## Figures and Tables

**Table 1:** The characteristics of included patients.

Variables	Total Cohort (*n* = 1203)	Patients with Follow-Up Visit (*n* = 509)		
		V1	V2	*p*-Value
Male	963 (80.0)	423 (83.1)		
Age (years)	61.1 (53.0, 69.0)	61.0 (53.4, 60.6)		
BMI (kg/m^2^)	25.8 (23.4, 27.8)	25.7 (22.8, 27.8)		
Killip (II- IV)	153 (12.7)	41 (8.1)		
LVEF (%)	55.0 (50.0, 60.0)	56.0 (50.0, 60.0)	60.0 (55.0, 63.0)	<0.001
LVEF < 40%	68 (5.7)	24 (4.7)	12 (2.4)	
MVD	883 (73.4)	365 (71.7)		
PCI	833 (69.2)	359 (70.5)		
Medical history				
Current Smoker	875 (72.7)	371 (72.9)		
Hypertension	786 (65.3)	324 (63.7)		
Hyperlipemia	1117 (92.9)	468 (91.9)		
Diabetes Mellitus	416 (34.6)	177 (34.8)		
Previous Stroke	184 (15.3)	79 (15.5)		
CKD	95 (7.9)	25 (4.9)		
PAD	73 (6.1)	35 (6.9)		
Previous MI	220 (18.3)	98 (19.3)		
Laboratory indexes				
TMAO (µmol/L)	6.6 (4.0, 11.6)	6.7 (4.0, 11.5)	12.7 (8.1, 20.4)	<0.001
Choline (µmol/L)	1.2 (1.0, 1.5)	1.2 (1.0, 1.5)	1.7 (1.4, 2.1)	<0.001
Betaine (µmol/L)	1.9 (1.5, 2.4)	1.9 (1.5, 2.4)	3.1 (2.4, 3.9)	<0.001
L-carnitine1 (µmol/L)	50.6 (42.5, 59.6)	51.2 (43.5, 60.7)	67.0 (51.5, 83.0)	<0.001
eGFR (mL/min/1.732 m^2^ *)	76.1 (64.1, 89.4)	75.9 (65.8, 90.4)	78.2 (65.3, 88.4)	0.219
ALT (IU/L)	32.0 (21.0, 52.0)	32.0 (20.0, 52.0)		
AST (IU/L)	90.0 (44.0, 191.0)	87.0 (44.0, 187.0)		
Baseline cTnI (ng/mL)	1.0 (0.1, 5.5)	1.1 (0.1, 6.2)		
Peak cTnI (ng/mL)	9.6 (2.1, 27.2)	9.3 (2.0, 24.6)		
Baseline NT-proBNP (ng/mL)	301.8 (83.1, 1007.0)	252.5 (70.4, 749.2)		
Peak NT-proBNP (ng/mL)	1186.0 (469.6, 2776.0)	1056.0 (429.4, 2186.0)		
hsCRP (mg/L)	5.5 (1.8, 11.0)	5.4 (1.8, 10.8)	1.2 (0.5, 2.8)	<0.001
LDL-C (mmol/L)	2.6 (2.0, 3.2)	2.6 (2.0, 3.2)	1.8 (1.5, 2.3)	<0.001
GRACE score	108.0 (89.0, 127.0)	107.0 (90.0, 125.0)		
Medication at discharge				
Aspirin	1142 (94.9)	484 (95.1)		
Ticagrelor	565 (47.0)	251 (49.3)		
Clopidogrel	607 (50.5)	255 (50.1)		
ACEI/ARB	830 (69.0)	356 (69.9)		
Βeta Blocker	1008 (83.8)	443 (87.0)		
Statins	1139 (94.7)	497 (97.6)		
Adverse outcomes				
Death	84 (7.0)	24 (4.7)		
reMI	71 (5.9)	27 (5.3)		
reHF	22 (1.8)	12 (2.4)		
Revascularization	195 (16.2)	71 (13.9)		
Stroke	41(3.4)	18 (3.5)		
MACE	343 (28.5)	125 (24.6)		

Continuous variables are presented as medians (25th−75th percentiles), and categorical variables are reported as counts (%). ACEIs/ARBs indicates angiotensin-converting enzyme inhibitors/angiotensin receptor blockers; ALT, alanine aminotransferase; AST, aspartate aminotransferase; BMI, body mass index; CKD, chronic kidney disease; cTnI, cardiac troponin I; GRACE, the Global Registry of Acute Coronary Events; HF, heart failure; hsCRP, high-sensitivity C-reactive protein; LDL-C, low-density lipoprotein cholesterol; LVEF, left ventricular ejection fraction; MACE, major adverse cardiovascular event; MI, myocardial infarction; MVD, multiple vessels disease; reHF, rehospitalization caused by heart failure; reMI, recurrent myocardial infarction; STMEI, ST-segment elevation myocardial infarction; NT-proBNP, N-terminal pro B-type natriuretic peptide; STMEI, ST-segment elevation myocardial infarction; PAD, peripheral artery disease; PCI, percutaneous coronary intervention; TMAO, trimethylamine-N-oxide. * Estimated glomerular filtration rate (eGFR) was calculated according to the Modification of Diet in Renal Disease formula.

**Table 2:** Association between TMAO levels at enrollment and all endpoints.

Endpoint	Group	Event (*n*.%)	Crude HR (95%CI)	*p*-Value	Adjusted HR (95%CI) *	*p*-Value
MACE						
	≤median	149 (24.8)	[1]		[1]	
	>median	194 (32.2)	1.39 (1.12–1.72)	0.003	1.27 (1.01–1.60)	0.040
	Tertile 1	102 (25.4)	[1]		[1]	
	Tertile 2	97 (24.2)	0.95 (0.72–1.26)	0.743	0.89 (0.67–1.19)	0.429
	Tertile 3	144 (35.9)	1.54 (1.20–1.99)	0.001	1.35 (1.02–1.78)	0.033
	Trend. test	343 (28.5)	1.26 (1.10–1.44)	0.001	1.18 (1.02–1.36)	0.025
All-cause death						
	≤median	31 (5.2)	[1]		[1]	
	>median	53 (8.8)	1.76 (1.13–2.74)	0.013	1.22 (0.73–2.04)	0.441
	Tertile 1	16 (4.0)	[1]		[1]	
	Tertile 2	23 (5.7)	1.45 (0.77–2.74)	0.255	1.44 (0.68–3.02)	0.341
	Tertile 3	45 (11.2)	2.97 (1.68–5.26)	< 0.001	2.06 (1.01–4.19)	0.047
	Trend. test	84 (7.0)	1.78 (1.34–2.36)	< 0.001	1.43 (1.02–2.01)	0.036
reMI						
	≤median	24 (4.0)	[1]		[1]	
	>median	47 (7.8)	2.04 (1.25–3.33)	0.005	2.01 (1.20–3.37)	0.008
	Tertile 1	19 (4.7)	[1]		[1]	
	Tertile 2	17 (4.2)	0.90 (0.47–1.73)	0.756	0.93 (0.48–1.81)	0.836
	Tertile 3	35 (8.7)	1.96 (1.12–3.43)	0.018	1.95 (1.08–3.53)	0.027
	Trend. test	71 (5.9)	1.46 (1.09–1.96)	0.012	1.44 (1.06–1.97)	0.021
reHF						
	≤median	8 (1.3)	[1]		[1]	
	>median	14 (2.3)	1.81 (0.76–4.31)	0.182	1.02 (0.39–2.64)	0.968
	Tertile 1	7 (1.7)	[1]		[1]	
	Tertile 2	4 (1.0)	0.57 (0.17–1.96)	0.375	0.37 (0.09–1.45)	0.152
	Tertile 3	11 (2.7)	1.67 (0.65–4.31)	0.288	0.97 (0.34–2.80)	0.961
	Trend. test	22 (1.8)	1.36 (0.81–2.30)	0.247	1.02 (0.57–1.82)	0.940
Stroke						
	≤median	20 (3.3)	[1]		[1]	
	>median	21 (3.5)	1.09 (0.59–2.01)	0.782	0.85 (0.44–1.66)	0.638
	Tertile 1	16 (4.0)	[1]		[1]	
	Tertile 2	13 (3.2)	0.83 (0.40–1.72)	0.607	0.77 (0.36–1.64)	0.500
	Tertile 3	12 (3.0)	0.80 (0.38–1.70)	0.566	0.52 (0.23–1.19)	0.121
	Trend. test	41 (3.4)	0.89 (0.61–1.30)	0.556	0.72 (0.48–1.09)	0.120
Revascularization						
	≤median	89 (14.8)	[1]		[1]	
	>median	106 (17.6)	1.25 (0.94–1.65)	0.125	1.3 (0.97–1.75)	0.083
	Tertile 1	62 (15.5)	[1]		[1]	
	Tertile 2	61 (15.2)	1.00 (0.70–1.42)	0.978	0.96 (0.67–1.38)	0.822
	Tertile 3	72 (18.0)	1.24 (0.89–1.75)	0.209	1.25 (0.88–1.79)	0.210
	Trend. test	195 (16.2)	1.12 (0.94–1.33)	0.205	1.12 (0.94–1.35)	0.212

HR, hazard ratio; MACE, major adverse cardiovascular event; reHF, rehospitalization caused by heart failure; reMI, recurrent myocardial infarction; TMAO, trimethylamine-N-oxide. * Adjusted for the variables with *p* < 0.1 in the univariable models, including age, hypertension, diabetes, peripheral artery disease, chronic kidney disease, and previous history of stroke and MI, Killip II-IV, the Global Registry of Acute Coronary Events risk score, multiple vessels disease, percutaneous coronary intervention, and the peak value of cardiac troponin I and N-terminal pro B-type natriuretic peptide during hospitalization, as well as estimated glomerular filtration rate and left ventricular ejection fraction.

**Table 3:** Association between TMAO levels of V1 and V2 and all endpoints.

Endpoint	Group	Event (*n*.%)	Crude HR (95%CI)	*p*-Value	Adjusted HR (95%CI) *	*p*-Value
MACE						
	L/L	33 (21.3)	[1]		[1]	
	L/H	17 (17.2)	0.79 (0.44–1.42)	0.433	1.01 (0.55–1.86)	0.980
	H/L	22 (22.2)	1.04 (0.61–1.79)	0.875	1.20 (0.67–2.16)	0.547
	H/H	47 (30.1)	1.51 (0.97–2.36)	0.069	1.60 (0.98–2.61)	0.061
	Trend. test	119 (23.4)	1.17 (1.01–1.36)	0.041	1.18 (1.00–1.38)	0.047
	Group 1	50 (19.7)	[1]		[1]	
	Group 2	22 (22.2)	1.14 (0.69–1.88)	0.614	1.20 (0.69–2.06)	0.520
	Group 3	47 (30.1)	1.65 (1.11–2.45)	0.014	1.59 (1.03–2.46)	0.034
	Trend. test	119 (23.4)	1.28 (1.05–1.57)	0.015	1.26 (1.02–1.57)	0.036
All-cause death						
	L/L	5 (3.2)	[1]		[1]	
	L/H	4 (4.0)	1.26 (0.34–4.68)	0.734	1.95 (0.40–9.56)	0.410
	H/L	4 (4.0)	1.28 (0.34–4.78)	0.709	3.14 (0.62–15.94)	0.168
	H/H	11 (7.1)	2.28 (0.79–6.57)	0.126	2.55 (0.62–10.49)	0.196
	Trend. test	24 (4.7)	1.32 (0.93–1.85)	0.117	1.30 (0.87–1.94)	0.196
	Group 1	9 (3.5)	[1]		[1]	
	Group 2	4 (4.0)	1.17 (0.36–3.79)	0.797	1.19 (0.34–4.15)	0.786
	Group 3	11 (7.1)	2.07 (0.86–5.01)	0.104	1.66 (0.60–4.59)	0.330
	Trend. test	24 (4.7)	1.45 (0.92–2.26)	0.107	1.29 (0.77–2.15)	0.327
reMI						
	L/L	5 (3.2)	[1]		[1]	
	L/H	2 (2.0)	0.63 (0.12–3.25)	0.582	0.78 (0.15–4.20)	0.774
	H/L	8 (8.1)	2.62 (0.86–8.01)	0.091	3.30 (1.00–10.84)	0.050
	H/H	8 (5.1)	1.65 (0.54–5.06)	0.377	1.89 (0.58–6.16)	0.290
	Trend. test	23 (4.5)	1.27 (0.90–1.79)	0.178	1.33 (0.93–1.91)	0.116
	Group 1	7 (2.8)	[1]		[1]	
	Group 2	8 (8.1)	3.06 (1.11–8.43)	0.031	3.33 (1.18–9.42)	0.023
	Group 3	8 (5.1)	1.93 (0.70–5.33)	0.204	2.55 (0.90–7.22)	0.078
	Trend. test	23 (4.5)	1.37 (0.87–2.17)	0.174	1.59 (0.99–2.55)	0.057
reHF						
	L/L	3 (1.9)	[1]		[1]	
	L/H	2 (2.0)	1.05 (0.18–6.29)	0.957	3.39 (0.19–59.02)	0.403
	H/L	2 (2.0)	1.06 (0.18–6.34)	0.950	0.23 (0.01–6.30)	0.384
	H/H	4 (2.6)	1.36 (0.30–6.07)	0.689	0.19 (0.02–1.94)	0.160
	Trend. test	11 (2.2)	1.10 (0.68–1.80)	0.692	0.54 (0.26–1.09)	0.087
	Group 1	5 (2.0)	[1]		[1]	
	Group 2	2 (2.0)	1.04 (0.20–5.35)	0.964	0.52 (0.06–4.44)	0.551
	Group 3	4 (2.6)	1.33 (0.36–4.96)	0.670	0.94 (0.16–5.49)	0.943
	Trend. test	11 (2.2)	1.15 (0.59–2.23)	0.677	0.93 (0.38–2.31)	0.883
Stroke						
	L/L	5 (3.2)	[1]		[1]	
	L/H	2 (2.0)	0.62 (0.12–3.20)	0.570	0.54 (0.1–3.00)	0.484
	H/L	4 (4.0)	1.28 (0.34–4.77)	0.713	1.12 (0.26–4.75)	0.880
	H/H	7 (4.5)	1.45 (0.46–4.56)	0.528	0.93 (0.23–3.74)	0.914
	Trend. test	18 (3.5)	1.18 (0.80–1.74)	0.402	1.02 (0.64–1.62)	0.946
	Group 1	7 (2.8)	[1]		[1]	
	Group 2	4 (4.0)	1.5 (0.44–5.13)	0.516	1.34 (0.38–4.75)	0.649
	Group 3	7 (4.5)	1.7 (0.60–4.85)	0.321	1.13 (0.35–3.71)	0.836
	Trend. test	18 (3.5)	1.3 (0.78–2.18)	0.314	1.07 (0.60–1.91)	0.818
Revascularization						
	L/L	21 (13.5)	[1]		[1]	
	L/H	8 (8.1)	0.58 (0.26–1.31)	0.190	0.71 (0.31–1.64)	0.429
	H/L	11 (11.1)	0.80 (0.39–1.66)	0.551	0.91 (0.41–2.01)	0.823
	H/H	29 (18.6)	1.42 (0.81–2.49)	0.221	1.90 (1.05–3.45)	0.035
	Trend. test	69 (13.6)	1.16 (0.95–1.41)	0.152	1.26 (1.03–1.55)	0.026
	Group 1	29 (11.4)	[1]		[1]	
	Group 2	11 (11.1)	0.96 (0.48–1.92)	0.911	1.12 (0.55–2.28)	0.764
	Group 3	29 (18.6)	1.70 (1.02–2.85)	0.042	2.21 (1.28–3.79)	0.004
	Trend. test	69 (13.6)	1.31 (1.00–1.70)	0.048	1.48 (1.12–1.96)	0.005

Patients were divided into four groups according to TMAO levels at the enrollment (V1) and follow-up visit (V2) relative to the median of each visit point (6.7 µmol/L and 12.7 µmol/L for V1 and V2, respectively). L/L, low V1 and low V2; L/H, low V1 and high V2; H/L, high V1 and low V2; H/H, high V1 and high V2. Group1: patients with TMAO levels below the median at V1; Group 2: patients with higher levels of TMAO at V1 and subsequently lower levels at V2; Group3: patients with both higher TMAO levels at V1 and V2. HR, hazard ratio; MACE, major adverse cardiovascular event; reHF, rehospitalization caused by heart failure; reMI, recurrent myocardial infarction; TMAO, trimethylamine-N-oxide. * Adjusted for the variables with *p* < 0.1 in the univariable models, including age, hypertension, diabetes, peripheral artery disease, chronic kidney disease, and previous history of stroke and MI, Killip II-IV, the Global Registry of Acute Coronary Events risk score, multiple vessels disease, percutaneous coronary intervention, and the peak value of cardiac troponin I and N-terminal pro B-type natriuretic peptide during hospitalization, as well as estimated glomerular filtration rate and left ventricular ejection fraction at V2.

## Supplementary Materials

The following supporting information can be downloaded at: https://www.mdpi.com/article/10.3390/jcdd9110380/s1. Figure S1: The flowchart of the patient selection process; Figure S2: Continuous hazard ratios across TMAO levels at enrollment for major adverse cardiovascular event (A), all-cause death (B), myocardial infarction (C), rehospitalization caused by heart failure (D), stroke (E), and revascularization (F); Figure S3: Kaplan-Meier curve for cumulative event-free survival in groups stratified by choline tertile levels at enrollment. A: major adverse cardiovascular event; B: all-cause death; C: myocardial infarction; D: rehospitalization caused by heart failure; E: stroke; F: revascularization; Figure S4: Kaplan-Meier curve for cumulative event-free survival in groups stratified by betaine tertile levels at enrollment. A: major adverse cardiovascular event; B: all-cause death; C: myocardial infarction; D: rehospitalization caused by heart failure; E: stroke; F: revascularization; Figure S5: Kaplan-Meier curve for cumulative event-free survival in groups stratified by L-carnitine tertile levels at enrollment. A: major adverse cardiovascular event; B: all-cause death; C: myocardial infarction; D: rehospitalization caused by heart failure; E: stroke; F: revascularization; Figure S6: Kaplan-Meier curve for cumulative MACE-free survival in groups stratified by choline levels at enrollment (V1) and follow-up visit (V2). Patients were divided into three groups according to the respective median levels of choline at V1 and V2, namely Group 1 (patients with low choline levels at V1), Group 2 (patients with high choline levels at V1 and subsequently low levels at V2) and Group 3 (patients with high choline levels at V1 and V2). MACE, major adverse cardiovascular event; Table S1: Patient characteristics according to the median and tertile levels of TMAO at enrollment; Table S2: Patient characteristics according to median and tertile levels of choline at enrollment; Table S3: Patient characteristics according to the median and tertile levels of choline at enrollment; Table S4: Patient characteristics according to the median and tertile levels of L-carnitine at enrollment; Table S5: Differences in characteristics at baseline between patients with and without TMAO measurements at follow-up visit; Table S6: Association between choline levels at enrollment and all endpoints; Table S7: Association between betaine levels at enrollment and all endpoints; Table S8: Association between L-carnitine levels at enrollment and all endpoints; Table S9: Association between choline levels of V1 and V2 and all endpoints; Table S10: Association between betaine levels of V1 and V2 and all endpoints; Table S11: Association between L-carnitine levels of V1 and V2 and all endpoints.

## Abbreviations

AMI	Acute Myocardial Infarction
CHD	Coronary Heart Disease
CIs	Confidence Intervals
CKD	Chronic Kidney Disease
HRs	Hazard Ratios
HF	Heart Failure
IQR	Interquartile Range
MACE	Major Adverse Cardiovascular Events
PCI	Percutaneous Coronary Intervention
RCS	Restricted Cubic Spline
TMAO	Trimethylamine N-oxide
