# Supplementary material for: Association between the Changes in Trimethylamine N-Oxide-Related Metabolites and Prognosis of Patients with Acute Myocardial Infarction: A Prospective Study
Source: J Cardiovasc Dev Dis. 2022 Nov 4;9(11):380. doi: 10.3390/jcdd9110380 (PMC9694290; doi:10.3390/jcdd9110380)
Supplement: Supplementary file 1 [file jcdd-09-00380-s001.zip › jcdd-1993564-supplementary.pdf]

Table S1. Patient characteristics according to median and tertile levels of TMAO at enrollment.

Variables	Total (n = 1203)	Low (n = 601)	High (n = 602)	*P*-value
TMAO (μmol/L)	6.6 (4.0, 11.6)	4.0 (2.8, 5.2)	11.6 (8.5, 17.6)	< 0.001
Demographics				
Male	963 (80.0)	484 (80.5)	479 (79.6)	0.729
Age (years)	61.1 (53.0, 69.0)	60.0 (50.4, 67.0)	63.0 (55.0, 72.0)	< 0.001
BMI (kg/m^2)	25.8 (23.4, 27.8)	25.7 (23.3, 27.7)	25.8 (23.4, 28.1)	0.152
Killip (II- IV)	153 (12.7)	59 (9.8)	94 (15.6)	0.003
LVEF (%)	55.0 (50.0, 60.0)	56.0 (52.0, 60.0)	55.0 (48.0, 59.0)	0.037
LVEF < 40%	68 (5.7)	28 (4.7)	40 (6.6)	0.172
MVD	883 (73.4)	440 (73.2)	443 (73.6)	0.934
PCI	833 (69.2)	419 (69.7)	414 (68.8)	0.769
Medical history				
Current Smoker	875 (72.7)	434 (72.2)	441 (73.3)	0.733
Hypertension	786 (65.3)	381 (63.4)	405 (67.3)	0.176
Hyperlipemia	1117 (92.9)	550 (91.5)	567 (94.2)	0.092
Diabetes Mellitus	416 (34.6)	170 (28.3)	246 (40.9)	< 0.001
Previous Stroke	184 (15.3)	68 (11.3)	116 (19.3)	< 0.001
CKD	95 (7.9)	29 (4.8)	66 (11.0)	< 0.001
PAD	73 (6.1)	30 (5.0)	43 (7.1)	0.149
Previous MI	220 (18.3)	101 (16.8)	119 (19.8)	0.210
Laboratory indexes				
Choline (μmol/L)	1.2 (1.0, 1.5)	1.1 (0.9, 1.4)	1.2 (1.0, 1.6)	< 0.001
Betaine (μmol/L)	1.9 (1.5, 2.4)	1.8 (1.4, 2.2)	2.0 (1.6, 2.5)	< 0.001
L-carnitine (μmol/L)	50.6 (42.5, 59.6)	49.5 (41.5, 58.6)	51.8 (43.5, 61.1)	0.003
eGFR (ml/min/1.732m^{2*})	76.1 (64.1, 89.4)	79.4 (68.9, 92.5)	72.3 (59.5, 85.4)	< 0.001
ALT (IU/L)	32.0 (21.0, 52.0)	32.0 (21.0, 50.0)	33.0 (20.0, 56.0)	0.933
AST (IU/L)	90.0 (44.0, 191.0)	97.0 (43.8, 186.0)	84.0 (44.0, 195.0)	0.961
Baseline cTnI (ng/mL)	1.0 (0.1, 5.5)	1.2 (0.1, 6.3)	0.8 (0.1, 4.5)	0.014
Peak cTnI (ng/mL)	9.6 (2.1, 27.2)	11.1 (3.0, 27.8)	8.6 (1.4, 26.7)	0.035
Baseline NT-proBNP (ng/mL)	301.8 (83.1, 1007.0)	277.0 (76.8, 881.0)	329.6 (89.5, 1103.0)	0.073
Peak NT-proBNP (ng/mL)	1186.0 (469.6, 2776.0)	1075.0 (413.3, 2544.0)	1287.0 (499.9, 2948.0)	0.092
hsCRP (mg/L)	5.5 (1.8, 11.0)	6.2 (1.9, 11.2)	4.8 (1.7, 10.8)	0.022
LDL-C (mmol/L)	2.6 (2.0, 3.2)	2.7 (2.1, 3.3)	2.4 (1.8, 3.0)	< 0.001
GRACE score	108.0 (89.0, 127.0)	105.0 (86.0, 123.0)	111.0 (93.0, 131.0)	< 0.001
Medication at discharge				
Aspirin	1142 (94.9)	578 (96.2)	564 (93.7)	0.063
Ticagrelor	565 (47.5)	307 (51.4)	258 (43.5)	0.007
Clopidogrel	607 (51.0)	281 (47.1)	326 (55.0)	0.008
ACEI/ARB	830 (69.7)	415 (69.5)	415 (70.0)	0.910
Beta Blocker	1008 (84.7)	516 (86.4)	492 (83.0)	0.114
Statins	1139 (95.7)	579 (97.0)	560 (94.4)	0.043
Adverse outcomes				
Death	84 (7.0)	31 (5.2)	53 (8.8)	0.018
reMI	71 (5.9)	24 (4.0)	47 (7.8)	0.007
reHF	22 (1.8)	8 (1.3)	14 (2.3)	0.284
Revascularization	195 (16.2)	89 (14.8)	106 (17.6)	0.215
Stroke	41 (3.4)	20 (3.3)	21 (3.5)	1.000
MACE	343 (28.5)	149 (24.8)	194 (32.2)	0.005
**Variables.**	**Tertile 1 (n = 401)**	**Tertile 2 (n = 401)**	**Tertile 3 (n = 401)**	***P*-value**

TMAO (μmol/L)	3.2 (2.2, 4.0)	6.6 (5.6, 7.9)	15.1 (11.6, 22.5)	< 0.001
Demographics				
Male	319 (79.6)	334 (83.3)	310 (77.3)	0.101
Age (years)	59.0 (50.5, 67.0)	61.0 (52.0, 68.0)	64.0 (56.0, 73.0)	< 0.001
BMI (kg/m^2)	25.2 (23.1, 27.7)	25.8 (23.4, 28.0)	25.9 (23.5, 27.8)	0.093
Killip (II- IV)	36 (9.0)	42 (10.5)	75 (18.7)	< 0.001
LVEF (%)	55.0 (52.0, 60.0)	56.0 (50.0, 60.0)	55.0 (48.0, 59.0)	0.135
LVEF < 40%	18 (4.5)	17 (4.2)	33 (8.2)	0.023
MVD	287 (71.6)	291 (72.6)	305 (76.1)	0.319
PCI	282 (70.3)	284 (70.8)	267 (66.6)	0.364
Medical history				
Current Smoker	282 (70.3)	308 (76.8)	285 (71.1)	0.079
Hypertension	261 (65.1)	257 (64.1)	268 (66.8)	0.711
Hyperlipemia	370 (92.3)	368 (91.8)	379 (94.5)	0.275
Diabetes Mellitus	109 (27.2)	129 (32.2)	178 (44.4)	< 0.001
Previous Stroke	42 (10.5)	60 (15.0)	82 (20.4)	< 0.001
CKD	20 (5.0)	22 (5.5)	53 (13.2)	< 0.001
PAD	22 (5.5)	21 (5.2)	30 (7.5)	0.345
Previous MI	68 (17.0)	77 (19.2)	75 (18.7)	0.689
Laboratory indexes				
Choline (μmol/L)	1.1 (0.9, 1.3)	1.2 (1.0, 1.5)	1.3 (1.1, 1.6)	< 0.001
Betaine (μmol/L)	1.8 (1.4, 2.2)	1.9 (1.6, 2.4)	2.0 (1.6, 2.5)	< 0.001
L-carnitine (μmol/L)	49.5 (41.2, 58.3)	49.7 (42.6, 59.0)	52.5 (43.5, 62.0)	0.002
eGFR (ml/min/1.732m^{2}*)	80.2 (70.2, 94.2)	77.0 (65.4, 89.7)	69.3 (56.6, 82.3)	< 0.001
ALT (IU/L)	32.0 (21.5, 50.0)	33.0 (21.0, 52.0)	33.0 (19.0, 55.0)	0.875
AST (IU/L)	99.0 (44.0, 184.5)	90.0 (44.0, 194.8)	82.0 (44.0, 195.0)	0.982
Baseline cTnI (ng/mL)	1.5 (0.1, 6.9)	1.0 (0.2, 5.1)	0.7 (0.1, 4.4)	0.008
Peak cTnI (ng/mL)	12.7 (3.6, 29.8)	7.5 (1.9, 24.3)	9.5 (1.4, 27.9)	0.004
Baseline NT-proBNP (ng/mL)	296.7 (87.0, 904.2)	274.5 (77.0, 858.7)	338.3 (86.0, 1262.0)	0.180
Peak NT-proBNP (ng/mL)	1135.0 (442.8, 2544.0)	1004.0 (405.3, 2307.0)	1478.0 (525.0, 3217.0)	0.005
hsCRP (mg/L)	6.7 (2.0, 11.2)	5.0 (1.7, 10.8)	5.0 (1.7, 11.0)	0.058
LDL-C (mmol/L)	1.1 (0.9, 1.2)	1.0 (0.9, 1.2)	1.0 (0.9, 1.2)	0.099
GRACE score	104.0 (85.8, 123.0)	107.0 (88.0, 124.0)	114.0 (95.0, 136.0)	< 0.001
Medication at discharge				
Aspirin	385 (96.0)	384 (95.8)	373 (93.0)	0.135
Ticagrelor	209 (52.4)	191 (48.0)	165 (42.0)	0.013
Clopidogrel	185 (46.4)	203 (51.0)	219 (55.7)	0.031
ACEI/ARB	275 (68.9)	286 (71.9)	269 (68.4)	0.526
Beta Blocker	346 (86.7)	338 (84.9)	324 (82.4)	0.245
Statins	387 (97.0)	376 (94.5)	376 (95.7)	0.214
Adverse outcomes				
Death	16 (4.0)	23 (5.7)	45 (11.2)	< 0.001
reMI	19 (4.7)	17 (4.2)	35 (8.7)	0.013
reHF	7 (1.7)	4 (1.0)	11 (2.7)	0.180
Revascularization	62 (15.5)	61 (15.2)	72 (18.0)	0.507
Stroke	16 (4.0)	13 (3.2)	12 (3.0)	0.720
MACE	102 (25.4)	97 (24.2)	144 (35.9)	< 0.001

Continuous variables are presented as medians (25–75th percentiles), and categorical variables are reported as counts (%). ACEIs/ARBs indicates angiotensin-converting enzyme inhibitors/angiotensin receptor blockers; ALT, alanine aminotransferase; AST, aspartate aminotransferase; BMI, body

mass index; CKD, chronic kidney disease; cTnI, cardiac troponin I; GRACE, the Global Registry of Acute Coronary Events; HF, heart failure; hsCRP, high-sensitivity C-reactive protein; LDL-C, low-density lipoprotein cholesterol; LVEF, left ventricular ejection fraction; MACE, major adverse cardiovascular event; MI, myocardial infarction; MVD, multiple vessels disease; reHF, rehospitalization caused by heart failure; reMI, recurrent myocardial infarction; STMEI, ST-segment elevation myocardial infarction; NT-proBNP, N-terminal pro B-type natriuretic peptide; STMEI, ST-segment elevation myocardial infarction; PAD, peripheral artery disease; PCI, percutaneous coronary intervention; TMAO, trimethylamine-N-oxide. *estimated glomerular filtration rate (eGFR) was calculated according to the Modification of Diet in Renal Disease formula.

Table S2. Patient characteristics according to median and tertile levels of choline at enrollment.

Variables	Total (n = 1203)	Low (n = 601)	High (n = 602)	*P*-value
Choline (μmol/L)	1.2 (1.0, 1.5)	1.0 (0.8, 1.1)	1.5 (1.3, 1.7)	< 0.001
Demographics				
Male	963 (80.0)	473 (78.7)	490 (81.4)	0.273
Age (years)	61.1 (53.0, 69.0)	60.0 (51.0, 67.0)	63.0 (55.0, 71.0)	< 0.001
BMI (kg/m^2)	25.8 (23.4, 27.8)	25.6 (23.3, 27.7)	25.9 (23.4, 28.1)	0.024
Killip (II- IV)	153 (12.7)	62 (10.3)	91 (15.1)	0.016
LVEF (%)	55.0 (50.0, 60.0)	56.0 (50.0, 60.0)	55.0 (50.0, 59.0)	0.204
LVEF < 40%	68 (5.7)	29 (4.8)	39 (6.5)	0.264
MVD	883 (73.4)	444 (73.9)	439 (72.9)	0.757
PCI	833 (69.2)	428 (71.2)	405 (67.3)	0.156
Medical history				
Current Smoker	875 (72.7)	432 (71.9)	443 (73.6)	0.548
Hypertension	786 (65.3)	369 (61.4)	417 (69.3)	0.005
Hyperlipemia	1117 (92.9)	551 (91.7)	566 (94.0)	0.144
Diabetes Mellitus	416 (34.6)	183 (30.4)	233 (38.7)	0.003
Previous Stroke	184 (15.3)	75 (12.5)	109 (18.1)	0.009
CKD	95 (7.9)	32 (5.3)	63 (10.5)	0.001
PAD	73 (6.1)	33 (5.5)	40 (6.6)	0.473
Previous MI	220 (18.3)	97 (16.1)	123 (20.4)	0.064
Laboratory indexes				
TMAO (μmol/L)	6.6 (4.0, 11.6)	5.4 (3.4, 9.3)	8.0 (5.0, 14.1)	< 0.001
Betaine (μmol/L)	1.9 (1.5, 2.4)	1.7 (1.4, 2.1)	2.1 (1.7, 2.7)	< 0.001
L-carnitine (μmol/L)	50.6 (42.5, 59.6)	46.9 (40.0, 55.1)	54.6 (45.9, 64.3)	< 0.001
eGFR (ml/min/1.732m^{2*})	76.1 (64.1, 89.4)	79.3 (68.5, 91.2)	72.5 (59.4, 86.2)	< 0.001
ALT (IU/L)	32.0 (21.0, 52.0)	32.0 (22.0, 50.0)	33.0 (19.0, 53.0)	0.364
AST (IU/L)	90.0 (44.0, 191.0)	93.0 (44.0, 184.8)	87.0 (43.8, 197.0)	0.961
Baseline cTnI (ng/mL)	1.0 (0.1, 5.5)	1.1 (0.1, 6.1)	0.9 (0.1, 5.0)	0.385
Peak cTnI (ng/mL)	9.6 (2.1, 27.2)	11.5 (3.1, 28.2)	7.7 (1.5, 26.3)	0.012
Baseline NT-proBNP (ng/mL)	301.8 (83.1, 1007.0)	273.7 (76.4, 840.0)	339.8 (93.5, 1141.0)	0.023
Peak NT-proBNP (ng/mL)	1186.0 (469.6, 2776.0)	1080.0 (468.0, 2505.0)	1269.0 (469.7, 2956.0)	0.109
hsCRP (mg/L)	5.5 (1.8, 11.0)	5.1 (1.8, 11.0)	6.0 (2.0, 11.2)	0.326
LDL-C (mmol/L)	2.6 (2.0, 3.2)	2.7 (2.0, 3.3)	2.5 (1.9, 3.1)	0.003
GRACE score	108.0 (89.0, 127.0)	105.0 (87.0, 124.0)	110.0 (91.0, 130.0)	< 0.001
Medication at discharge				
Aspirin	1142 (94.9)	575 (95.7)	567 (94.2)	0.097
Ticagrelor	565 (47.5)	302 (50.5)	263 (44.4)	0.041
Clopidogrel	607 (51.0)	291 (48.7)	316 (53.4)	0.117
ACEI/ARB	830 (69.7)	411 (68.7)	419 (70.8)	0.480
Beta Blocker	1008 (84.7)	525 (87.8)	483 (81.6)	0.004
Statins	1139 (95.7)	574 (96.0)	565 (95.4)	0.747
Adverse outcomes				
Death	84 (7.0)	30 (5.0)	54 (9.0)	0.009
reMI	71 (5.9)	35 (5.8)	36 (6.0)	1.000
reHF	22 (1.8)	5 (0.8)	17 (2.8)	0.018
Revascularization	195 (16.2)	101 (16.8)	94 (15.6)	0.630
Stroke	41 (3.4)	16 (2.7)	25 (4.2)	0.206
MACE	343 (28.5)	155 (25.8)	188 (31.2)	0.043
**Variables**	**Tertile 1 (n = 401)**	**Tertile 2 (n = 401)**	**Tertile 3 (n = 401)**	***P*-value**

Choline (μmol/L)	0.9 (0.8, 1.0)	1.2 (1.1, 1.2)	1.6 (1.5, 1.9)	< 0.001
Demographics				
Male	95 (23.7)	73 (18.2)	72 (18.0)	0.071
Age (years)	60.0 (50.0, 67.0)	61.0 (54.0, 70.0)	63.0 (55.0, 72.0)	< 0.001
BMI (kg/m^2)	25.4 (23.1, 27.7)	25.9 (23.8, 28.0)	25.9 (22.9, 28.1)	0.041
Killip (II- IV)	39 (9.7)	52 (13.0)	62 (15.5)	0.050
LVEF (%)	56.0 (52.0, 60.0)	56.0 (50.0, 60.0)	55.0 (48.0, 59.0)	0.033
LVEF < 40%	17 (4.2)	20 (5.0)	31 (7.7)	0.079
MVD	296 (73.8)	298 (74.3)	289 (72.1)	0.752
PCI	278 (69.3)	287 (71.6)	268 (66.8)	0.347
Medical history				
Current Smoker	275 (68.6)	300 (74.8)	300 (74.8)	0.073
Hypertension	237 (59.1)	266 (66.3)	283 (70.6)	0.003
Hyperlipemia	375 (93.5)	361 (90.0)	381 (95.0)	0.019
Diabetes Mellitus	116 (28.9)	148 (36.9)	152 (37.9)	0.014
Previous Stroke	47 (11.7)	62 (15.5)	75 (18.7)	0.023
CKD	22 (5.5)	25 (6.2)	48 (12.0)	< 0.001
PAD	23 (5.7)	18 (4.5)	32 (8.0)	0.111
Previous MI	59 (14.7)	79 (19.7)	82 (20.4)	0.074
Laboratory indexes				
TMAO (μmol/L)	5.1 (3.2, 8.9)	6.6 (4.2, 11.3)	8.1 (5.0, 14.9)	< 0.001
Betaine (μmol/L)	1.6 (1.3, 2.0)	1.9 (1.5, 2.3)	2.2 (1.8, 2.9)	< 0.001
L-carnitine (μmol/L)	45.4 (38.7, 53.4)	51.7 (44.4, 59.6)	55.7 (46.2, 66.2)	< 0.001
eGFR (ml/min/1.732m^2*)	79.3 (68.3, 91.0)	77.5 (66.8, 91.7)	70.1 (56.8, 82.8)	< 0.001
ALT (IU/L)	32.0 (22.0, 49.0)	34.0 (22.0, 54.0)	31.0 (19.0, 53.0)	0.230
AST (IU/L)	101.0 (49.0, 181.0)	87.5 (43.0, 204.5)	80.0 (41.0, 181.0)	0.525
Baseline cTnI (ng/mL)	1.1 (0.1, 6.4)	1.0 (0.1, 5.0)	0.9 (0.1, 5.1)	0.805
Peak cTnI (ng/mL)	11.8 (3.4, 28.2)	10.0 (2.3, 27.7)	7.2 (1.3, 25.1)	0.042
Baseline NT-proBNP (ng/mL)	270.3 (67.1, 776.6)	309.6 (95.2, 900.9)	347.6 (92.8, 1331.0)	0.020
Peak NT-proBNP (ng/mL)	1056.0 (468.0, 2425.0)	1241.0 (481.0, 2712.0)	1344.0 (468.8, 3108.0)	0.165
hsCRP (mg/L)	4.7 (1.7, 10.6)	5.7 (1.9, 11.1)	6.2 (2.0, 11.3)	0.204
LDL-C (mmol/L)	2.7 (2.1, 3.3)	2.5 (1.9, 3.2)	2.5 (1.9, 3.0)	0.003
GRACE score	106.0 (88.0, 123.0)	107.0 (89.0, 129.0)	110.5 (91.0, 129.0)	0.011
Medication at discharge				
Aspirin	386 (96.3)	385 (96.0)	371 (92.5)	0.119
Ticagrelor	207 (51.9)	192 (48.2)	166 (42.2)	0.023
Clopidogrel	188 (47.1)	200 (50.3)	219 (55.7)	0.050
ACEI/ARB	272 (68.2)	280 (70.4)	278 (70.7)	0.697
Beta Blocker	348 (87.2)	338 (84.9)	322 (81.9)	0.117
Statins	384 (96.2)	380 (95.5)	375 (95.4)	0.816
Adverse outcomes				
Death	16 (4.0)	24 (6.0)	44 (11.0)	< 0.001
reMI	27 (6.7)	20 (5.0)	24 (6.0)	0.575
reHF	4 (1.0)	5 (1.2)	13 (3.2)	0.034
Revascularization	71 (17.7)	73 (18.2)	51 (12.7)	0.066
Stroke	10 (2.5)	13 (3.2)	18 (4.5)	0.290
MACE	104 (25.9)	116 (28.9)	123 (30.7)	0.323

Continuous variables are presented as medians (25–75th percentiles), and categorical variables are reported as counts (%). ACEIs/ARBs indicates angiotensin-converting enzyme inhibitors/angiotensin receptor blockers; ALT, alanine aminotransferase; AST, aspartate aminotransferase; BMI, body mass index; CKD, chronic kidney disease; cTnI, cardiac troponin I; GRACE, the Global Registry of Acute Coronary Events; HF, heart failure; hsCRP, high-sensitivity C-reactive protein; LDL-C, low-density lipoprotein cholesterol; LVEF, left ventricular ejection fraction; MACE, major adverse

cardiovascular event; MI, myocardial infarction; MVD, multiple vessels disease; reHF, rehospitalization caused by heart failure; reMI, recurrent myocardial infarction; STMEI, ST-segment elevation myocardial infarction; NT-proBNP, N-terminal pro B-type natriuretic peptide; STMEI, ST-segment elevation myocardial infarction; PAD, peripheral artery disease; PCI, percutaneous coronary intervention; TMAO, trimethylamine-N-oxide. *estimated glomerular filtration rate (eGFR) was calculated according to the Modification of Diet in Renal Disease formula.

Table S3. Patient characteristics according to median and tertile levels of betaine at enrollment.

Variables	Total (n = 1203)	Low (n = 601)	High (n = 602)	*P*-value
Betaine (μmol/L)	1.9 (1.5, 2.4)	1.5 (1.2, 1.7)	2.4 (2.1, 2.9)	< 0.001
Demographics				
Male	963 (80.0)	475 (79.0)	488 (81.1)	0.419
Age (years)	61.1 (53.0, 69.0)	60.0 (51.0, 68.0)	63.0 (55.0, 70.8)	< 0.001
BMI (kg/m^2)	25.8 (23.4, 27.8)	25.8 (23.4, 28.0)	25.8 (23.3, 27.7)	0.238
Killip (II- IV)	153 (12.7)	79 (13.1)	74 (12.3)	0.721
LVEF (%)	55.0 (50.0, 60.0)	55.0 (50.0, 60.0)	55.0 (50.0, 60.0)	0.860
LVEF < 40%	68 (5.7)	30 (5.0)	38 (6.3)	0.386
MVD	883 (73.4)	443 (73.7)	440 (73.1)	0.858
PCI	833 (69.2)	447 (74.4)	386 (64.1)	< 0.001
Medical history				
Current Smoker	875 (72.7)	434 (72.2)	441 (73.3)	0.733
Hypertension	786 (65.3)	387 (64.4)	399 (66.3)	0.531
Hyperlipemia	1117 (92.9)	551 (91.7)	566 (94.0)	0.144
Diabetes Mellitus	416 (34.6)	225 (37.4)	191 (31.7)	0.043
Previous Stroke	184 (15.3)	88 (14.6)	96 (15.9)	0.583
CKD	95 (7.9)	45 (7.5)	50 (8.3)	0.675
PAD	73 (6.1)	25 (4.2)	48 (8.0)	0.008
Previous MI	220 (18.3)	88 (14.6)	132 (21.9)	0.001
Laboratory indexes				
TMAO (μmol/L)	6.6 (4.0, 11.6)	6.1 (3.7, 11.3)	7.3 (4.4, 12.3)	0.002
Choline (μmol/L)	1.2 (1.0, 1.5)	1.1 (0.9, 1.3)	1.3 (1.1, 1.6)	< 0.001
L-carnitine (μmol/L)	50.6 (42.5, 59.6)	49.5 (41.5, 58.6)	51.8 (43.5, 61.1)	0.003
eGFR (ml/min/1.732m^{2*})	50.6 (42.5, 59.6)	47.6 (39.8, 54.6)	55.2 (45.8, 64.7)	< 0.001
ALT (IU/L)	32.0 (21.0, 52.0)	32.0 (21.0, 52.0)	33.0 (21.0, 51.8)	0.758
AST (IU/L)	90.0 (44.0, 191.0)	93.5 (44.0, 201.2)	87.0 (44.0, 181.8)	0.424
Baseline cTnI (ng/mL)	76.1 (64.1, 89.4)	76.1 (64.0, 89.7)	76.4 (64.4, 88.4)	0.983
Peak cTnI (ng/mL)	1.0 (0.1, 5.5)	1.1 (0.1, 6.7)	0.9 (0.1, 4.4)	0.159
Baseline NT-proBNP (ng/mL)	9.6 (2.1, 27.2)	13.5 (3.2, 31.1)	6.7 (1.5, 22.3)	< 0.001
Peak NT-proBNP (ng/mL)	301.8 (83.1, 1007.0)	276.1 (71.0, 919.1)	338.9 (98.8, 1063.0)	0.069
hsCRP (mg/L)	1186.0 (469.6, 2776.0)	1210.0 (461.8, 2842.0)	1129.0 (479.2, 2663.0)	0.741
LDL-C (mmol/L)	5.5 (1.8, 11.0)	6.7 (2.3, 11.2)	4.5 (1.6, 10.8)	< 0.001
GRACE score	2.6 (2.0, 3.2)	2.7 (2.1, 3.3)	2.4 (1.8, 3.1)	< 0.001
Medication at discharge				
Aspirin	1142 (94.9)	577 (96.0)	565 (93.9)	0.209
Ticagrelor	565 (47.5)	302 (50.8)	263 (44.1)	0.024
Clopidogrel	607 (51.0)	285 (48.0)	322 (54.0)	0.043
ACEI/ARB	830 (69.7)	413 (69.5)	417 (70.0)	0.919
Beta Blocker	1008 (84.7)	512 (86.2)	496 (83.2)	0.179
Statins	1139 (95.7)	561 (94.4)	578 (97.0)	0.044
Adverse outcomes				
Death	84 (7.0)	44 (7.3)	40 (6.6)	0.728
reMI	71 (5.9)	33 (5.5)	38 (6.3)	0.630
reHF	22 (1.8)	8 (1.3)	14 (2.3)	0.284
Revascularization	195 (16.2)	100 (16.6)	95 (15.8)	0.745
Stroke	41 (3.4)	18 (3.0)	23 (3.8)	0.529
MACE	343 (28.5)	175 (29.1)	168 (27.9)	0.688
**Variables**	**Tertile 1 (n = 401)**	**Tertile 2 (n = 401)**	**Tertile 3 (n = 401)**	***P*-value**

Betaine (μmol/L)	1.3 (1.1, 1.5)	1.9 (1.8, 2.0)	2.7 (2.4, 3.1)	< 0.001
Demographics				
Male	314 (78.3)	320 (79.8)	329 (82.0)	0.411
Age (years)	60.0 (49.7, 67.0)	60.0 (53.0, 68.0)	64.8 (56.0, 72.0)	< 0.001
BMI (kg/m^2)	26.1 (23.3, 28.4)	25.8 (23.7, 27.7)	25.5 (22.9, 27.7)	0.059
Killip (II- IV)	53 (13.2)	52 (13.0)	48 (12.0)	0.854
LVEF (%)	56.0 (50.0, 60.0)	55.0 (48.0, 59.0)	56.0 (50.0, 60.0)	0.282
LVEF < 40%	17 (4.2)	27 (6.7)	24 (6.0)	0.292
MVD	290 (72.3)	308 (76.8)	285 (71.1)	0.154
PCI	296 (73.8)	287 (71.6)	250 (62.3)	< 0.001
Medical history				
Current Smoker	296 (73.8)	282 (70.3)	297 (74.1)	0.413
Hypertension	253 (63.1)	265 (66.1)	268 (66.8)	0.500
Hyperlipemia	368 (91.8)	378 (94.3)	371 (92.5)	0.372
Diabetes Mellitus	159 (39.7)	130 (32.4)	127 (31.7)	0.032
Previous Stroke	59 (14.7)	60 (15.0)	65 (16.2)	0.820
CKD	27 (6.7)	35 (8.7)	33 (8.2)	0.552
PAD	15 (3.7)	27 (6.7)	31 (7.7)	0.048
Previous MI	50 (12.5)	80 (20.0)	90 (22.4)	< 0.001
Laboratory indexes				
TMAO (μmol/L)	5.7 (3.5, 10.5)	6.6 (4.3, 12.0)	7.5 (4.4, 12.8)	< 0.001
Choline (μmol/L)	1.0 (0.8, 1.3)	1.2 (1.0, 1.4)	1.4 (1.1, 1.7)	< 0.001
L-carnitine (μmol/L)	46.3 (37.7, 53.8)	51.6 (43.7, 59.6)	55.5 (46.0, 64.8)	< 0.001
eGFR (ml/min/1.732m^{2*})	75.6 (63.8, 89.0)	78.0 (65.7, 89.8)	74.3 (63.9, 87.9)	0.203
ALT (IU/L)	32.0 (21.0, 52.0)	33.0 (22.0, 54.0)	33.0 (19.0, 50.0)	0.421
AST (IU/L)	95.0 (44.0, 201.5)	99.0 (46.0, 193.2)	83.0 (43.0, 175.0)	0.395
Peak cTnI (ng/mL)	14.6 (4.0, 32.1)	8.2 (2.5, 27.2)	6.7 (1.1, 21.6)	< 0.001
Baseline NT-proBNP (ng/mL)	300.3 (74.8, 1037.0)	300.0 (80.1, 843.0)	306.9 (100.3, 1146.0)	0.631
Peak NT-proBNP (ng/mL)	1338.0 (481.9, 2873.0)	1031.0 (429.3, 2611.0)	1133.0 (481.0, 2586.0)	0.455
hsCRP (mg/L)	7.1 (2.5, 11.5)	5.3 (1.8, 11.1)	4.5 (1.5, 10.6)	0.001
LDL-C (mmol/L)	2.8 (2.2, 3.4)	2.6 (2.0, 3.2)	2.4 (1.7, 3.0)	< 0.001
GRACE score	105.0 (84.0, 125.0)	106.0 (88.0, 126.2)	111.0 (96.0, 129.0)	0.002
Medication at discharge				
Aspirin	388 (96.8)	382 (95.3)	372 (92.8)	0.026
Ticagrelor	209 (52.8)	183 (46.1)	173 (43.6)	0.027
Clopidogrel	185 (46.7)	206 (51.9)	216 (54.4)	0.087
ACEI/ARB	272 (68.7)	276 (69.5)	282 (71.0)	0.767
Beta Blocker	348 (87.9)	335 (84.4)	325 (81.9)	0.061
Statins	374 (94.4)	378 (95.2)	387 (97.5)	0.090
Adverse outcomes				
Death	27 (6.7)	28 (7.0)	29 (7.2)	0.962
reMI	19 (4.7)	29 (7.2)	23 (5.7)	0.321
reHF	6 (1.5)	3 (0.7)	13 (3.2)	0.026
Revascularization	67 (16.7)	65 (16.2)	63 (15.7)	0.929
Stroke	12 (3.0)	13 (3.2)	16 (4.0)	0.720
MACE	117 (29.2)	111 (27.7)	115 (28.7)	0.892

Continuous variables are presented as medians (25–75th percentiles), and categorical variables are reported as counts (%). ACEIs/ARBs indicates angiotensin-converting enzyme inhibitors/angiotensin receptor blockers; ALT, alanine aminotransferase; AST, aspartate aminotransferase; BMI, body mass index; CKD, chronic kidney disease; cTnI, cardiac troponin I; GRACE, the Global Registry of Acute Coronary Events; HF, heart failure; hsCRP, high-sensitivity C-reactive protein; LDL-C, low-density lipoprotein cholesterol; LVEF, left ventricular ejection fraction; MACE, major adverse cardiovascular event; MI, myocardial infarction; MVD, multiple vessels disease; reHF,

rehospitalization caused by heart failure; reMI, recurrent myocardial infarction; STMEI, ST-segment elevation myocardial infarction; NT-proBNP, N-terminal pro B-type natriuretic peptide; STMEI, ST-segment elevation myocardial infarction; PAD, peripheral artery disease; PCI, percutaneous coronary intervention; TMAO, trimethylamine-N-oxide. *estimated glomerular filtration rate (eGFR) was calculated according to the Modification of Diet in Renal Disease formula.

Table S4. Patient characteristics according to median and tertile levels of L-carnitine at enrollment.

Variables	Total (n = 1203)	Low (n = 601)	High (n = 602)	*P*-value
L-carnitine (μmol/L)	50.6 (42.5, 59.6)	42.5 (36.5, 46.7)	59.6 (55.0, 67.3)	< 0.001
Demographics				
Male	963 (80.0)	463 (77.0)	500 (83.1)	0.011
Age (years)	61.1 (53.0, 69.0)	61.0 (53.0, 69.0)	61.4 (54.0, 69.0)	0.723
BMI (kg/m²)	25.8 (23.4, 27.8)	25.4 (23.1, 27.7)	25.9 (23.5, 28.1)	0.003
Killip (II- IV)	153 (12.7)	77 (12.8)	76 (12.6)	0.991
LVEF (%)	55.0 (50.0, 60.0)	55.0 (50.0, 60.0)	55.0 (50.0, 60.0)	0.705
LVEF < 40%	68 (5.7)	31 (5.2)	37 (6.1)	0.537
MVD	883 (73.4)	434 (72.2)	449 (74.6)	0.387
PCI	833 (69.2)	426 (70.9)	407 (67.6)	0.243
Medical history				
Current Smoker	875 (72.7)	423 (70.4)	452 (75.1)	0.077
Hypertension	786 (65.3)	388 (64.6)	398 (66.1)	0.613
Hyperlipemia	1117 (92.9)	552 (91.8)	565 (93.9)	0.215
Diabetes Mellitus	416 (34.6)	225 (37.4)	191 (31.7)	0.043
Previous Stroke	184 (15.3)	87 (14.5)	97 (16.1)	0.479
CKD	95 (7.9)	53 (8.8)	42 (7.0)	0.281
PAD	73 (6.1)	28 (4.7)	45 (7.5)	0.054
Previous MI	220 (18.3)	90 (15.0)	130 (21.6)	0.004
Laboratory indexes				
TMAO (μmol/L)	6.6 (4.0, 11.6)	6.1 (3.8, 10.7)	7.3 (4.3, 12.5)	< 0.001
Choline (μmol/L)	1.2 (1.0, 1.5)	1.1 (0.9, 1.3)	1.3 (1.1, 1.6)	< 0.001
Betaine (μmol/L)	1.9 (1.5, 2.4)	1.7 (1.3, 2.1)	2.0 (1.7, 2.6)	< 0.001
eGFR (ml/min/1.732m²*)	76.1 (64.1, 89.4)	77.5 (64.5, 90.8)	75.1 (64.0, 87.5)	0.033
ALT (IU/L)	32.0 (21.0, 52.0)	31.0 (20.0, 50.0)	33.0 (21.0, 54.0)	0.141
AST (IU/L)	90.0 (44.0, 191.0)	88.0 (40.8, 185.2)	93.0 (47.0, 193.8)	0.288
Baseline cTnI (ng/mL)	1.0 (0.1, 5.5)	0.9 (0.1, 6.0)	1.0 (0.1, 5.2)	0.490
Peak cTnI (ng/mL)	9.6 (2.1, 27.2)	11.5 (2.8, 28.9)	8.4 (1.5, 25.3)	0.003
Baseline NT-proBNP (ng/mL)	301.8 (83.1, 1007.0)	288.6 (70.9, 955.3)	318.6 (96.1, 1047.0)	0.127
Peak NT-proBNP (ng/mL)	1186.0 (469.6, 2776.0)	1207.0 (495.5, 2892.0)	1146.0 (427.2, 2555.0)	0.243
hsCRP (mg/L)	5.5 (1.8, 11.0)	5.5 (1.9, 11.0)	5.5 (1.8, 11.2)	0.755
LDL-C (mmol/L)	2.6 (2.0, 3.2)	2.6 (2.0, 3.3)	2.5 (1.9, 3.1)	< 0.001
GRACE score	108.0 (89.0, 127.0)	109.0 (89.0, 128.0)	108.0 (89.0, 125.8)	0.547
Medication at discharge				
Aspirin	1142 (94.9)	570 (94.8)	572 (95.0)	0.731
Ticagrelor	565 (47.5)	281 (47.3)	284 (47.7)	0.951
Clopidogrel	607 (51.0)	303 (51.0)	304 (51.0)	1.000
ACEI/ARB	830 (69.7)	417 (70.2)	413 (69.3)	0.782
Beta Blocker	1008 (84.7)	509 (85.7)	499 (83.7)	0.389
Statins	1139 (95.7)	565 (95.1)	574 (96.3)	0.384
Adverse outcomes				
Death	84 (7.0)	38 (6.3)	46 (7.6)	0.433
reMI	71 (5.9)	31 (5.2)	40 (6.6)	0.331
reHF	22 (1.8)	13 (2.2)	9 (1.5)	0.516
Revascularization	195 (16.2)	93 (15.5)	102 (16.9)	0.540
Stroke	41 (3.4)	20 (3.3)	21 (3.5)	1.000
MACE	343 (28.5)	166 (27.6)	177 (29.4)	0.535

Variables	**Tertile 1 (n = 401)**	**Tertile 2 (n = 401)**	**Tertile 3 (n = 401)**	***P*-value**
L-carnitine (μmol/L)	38.8 (33.1, 42.5)	50.6 (47.9, 53.5)	64.6 (59.6, 71.3)	< 0.001

Demographics				
Male	297 (74.1)	330 (82.3)	336 (83.8)	0.001
Age (years)	62.0 (53.0, 69.0)	61.0 (53.0, 69.0)	62.0 (54.0, 68.0)	0.660
BMI (kg/m^2)	25.2 (22.9, 27.6)	25.9 (23.4, 28.1)	25.9 (23.6, 28.1)	0.003
Killip (II- IV)	49 (12.2)	51 (12.7)	53 (13.2)	0.914
LVEF (%)	55.0 (50.0, 60.0)	55.0 (50.0, 60.0)	56.0 (50.0, 60.0)	0.968
LVEF < 40%	18 (4.5)	25 (6.2)	25 (6.2)	0.466
MVD	291 (72.6)	300 (74.8)	292 (72.8)	0.733
PCI	287 (71.6)	289 (72.1)	257 (64.1)	0.023
Medical history				
Current Smoker	275 (68.6)	294 (73.3)	306 (76.3)	0.046
Hypertension	258 (64.3)	257 (64.1)	271 (67.6)	0.511
Hyperlipemia	369 (92.0)	371 (92.5)	377 (94.0)	0.521
Diabetes Mellitus	156 (38.9)	132 (32.9)	128 (31.9)	0.080
Previous Stroke	56 (14.0)	59 (14.7)	69 (17.2)	0.410
CKD	36 (9.0)	34 (8.5)	25 (6.2)	0.308
PAD	17 (4.2)	22 (5.5)	34 (8.5)	0.035
Previous MI	58 (14.5)	70 (17.5)	92 (22.9)	0.007
Laboratory indexes				
TMAO (μmol/L)	6.1 (3.7, 11.3)	6.6 (4.0, 11.1)	7.3 (4.5, 12.8)	0.011
Choline (μmol/L)	1.0 (0.8, 1.3)	1.1 (1.0, 1.4)	1.3 (1.1, 1.6)	< 0.001
Betaine (μmol/L)	1.7 (1.3, 2.1)	1.8 (1.5, 2.3)	2.1 (1.8, 2.7)	< 0.001
eGFR (ml/min/1.732m^{2}*)	77.0 (64.4, 91.0)	77.0 (65.2, 89.8)	74.2 (63.9, 86.8)	0.116
ALT (IU/L)	31.0 (20.0, 50.0)	32.0 (21.0, 50.0)	34.0 (21.0, 55.0)	0.381
AST (IU/L)	87.0 (41.0, 182.0)	90.0 (45.0, 203.0)	93.0 (43.8, 191.2)	0.760
Peak cTnI (ng/mL)	11.5 (2.9, 28.5)	9.8 (2.2, 27.6)	7.1 (1.4, 25.1)	0.017
Baseline NT-proBNP (ng/mL)	295.0 (61.5, 965.7)	302.5 (86.8, 852.0)	322.1 (92.9, 1203.0)	0.525
Peak NT-proBNP (ng/mL)	1274.0 (526.8, 2913.0)	1064.0 (402.5, 2629.0)	1196.0 (429.4, 2624.0)	0.239
hsCRP (mg/L)	5.1 (1.8, 10.7)	6.0 (2.0, 10.9)	5.4 (1.7, 11.4)	0.682
LDL-C (mmol/L)	2.7 (2.1, 3.3)	2.6 (2.0, 3.3)	2.4 (1.8, 3.0)	< 0.001
GRACE score	109.0 (90.0, 128.0)	107.0 (88.0, 126.0)	109.0 (90.0, 126.0)	0.445
Medication at discharge				
Aspirin	383 (95.5)	379 (94.5)	380 (94.8)	0.817
Ticagrelor	191 (48.2)	181 (45.7)	193 (48.5)	0.686
Clopidogrel	199 (50.3)	207 (52.3)	201 (50.5)	0.825
ACEI/ARB	271 (68.4)	280 (70.7)	279 (70.1)	0.771
Beta Blocker	342 (86.4)	335 (84.6)	331 (83.2)	0.456
Statins	377 (95.2)	377 (95.2)	385 (96.7)	0.469
Adverse outcomes				
Death	24 (6.0)	26 (6.5)	34 (8.5)	0.341
reMI	21 (5.2)	22 (5.5)	28 (7.0)	0.525
reHF	8 (2.0)	7 (1.7)	7 (1.7)	0.955
Revascularization	61 (15.2)	61 (15.2)	73 (18.2)	0.414
Stroke	11 (2.7)	14 (3.5)	16 (4.0)	0.619
MACE	104 (25.9)	112 (27.9)	127 (31.7)	0.189

Continuous variables are presented as medians (25–75th percentiles), and categorical variables are reported as counts (%). ACEIs/ARBs indicates angiotensin-converting enzyme inhibitors/angiotensin receptor blockers; ALT, alanine aminotransferase; AST, aspartate aminotransferase; BMI, body mass index; CKD, chronic kidney disease; cTnI, cardiac troponin I; GRACE, the Global Registry of Acute Coronary Events; HF, heart failure; hsCRP, high-sensitivity C-reactive protein; LDL-C, low-density lipoprotein cholesterol; LVEF, left ventricular ejection fraction; MACE, major adverse cardiovascular event; MI, myocardial infarction; MVD, multiple vessels disease; reHF, rehospitalization caused by heart failure; reMI, recurrent myocardial infarction; STMEI, ST-segment elevation

myocardial infarction; NT-proBNP, N-terminal pro B-type natriuretic peptide; STMEI, ST-segment elevation myocardial infarction; PAD, peripheral artery disease; PCI, percutaneous coronary intervention; TMAO, trimethylamine-N-oxide. *estimated glomerular filtration rate (eGFR) was calculated according to the Modification of Diet in Renal Disease formula.

Table S5. Differences in characteristics at baseline between patients with and without TMAO measurements at follow-up visit.

Variables	Total (n = 1203)	With V2 (n = 509)	Without V2 (n = 694)	*P*-value
Demographics				
Male	963 (80.0)	423 (83.1)	540 (77.8)	0.028
Age (years)	61.1 (53.0, 69.0)	61.0 (53.4, 68.0)	62.0 (53.0, 70.0)	0.263
BMI (kg/m^2)	25.8 (23.4, 27.8)	25.7 (22.8, 27.8)	25.8 (23.5, 28.0)	0.197
Killip (II- IV)	153 (12.7)	41 (8.1)	112 (16.1)	< 0.001
LVEF (%)	55.0 (50.0, 60.0)	56.0 (50.0, 60.0)	55.0 (50.0, 60.0)	0.147
LVEF < 40%	68 (5.7)	24 (4.7)	44 (6.3)	0.280
MVD	883 (73.4)	365 (71.7)	518 (74.6)	0.284
PCI	833 (69.2)	359 (70.5)	474 (68.3)	0.444
Medical history				
Current Smoker	875 (72.7)	371 (72.9)	504 (72.6)	0.971
Hypertension	786 (65.3)	324 (63.7)	462 (66.6)	0.323
Hyperlipemia	1117 (92.9)	468 (91.9)	649 (93.5)	0.352
Diabetes Mellitus	416 (34.6)	177 (34.8)	239 (34.4)	0.952
Previous Stroke	184 (15.3)	79 (15.5)	105 (15.1)	0.916
CKD	95 (7.9)	25 (4.9)	70 (10.1)	0.001
PAD	73 (6.1)	35 (6.9)	38 (5.5)	0.377
Previous MI	220 (18.3)	98 (19.3)	122 (17.6)	0.505
Laboratory indexes				
TMAO (μmol/L)	6.6 (4.0, 11.6)	6.7 (4.0, 11.5)	6.6 (4.0, 11.8)	0.891
Choline (μmol/L)	1.2 (1.0, 1.5)	1.2 (1.0, 1.5)	1.2 (1.0, 1.5)	0.500
Betaine (μmol/L)	1.9 (1.5, 2.4)	1.9 (1.5, 2.4)	1.9 (1.5, 2.4)	0.273
L-carnitine (μmol/L)	50.6 (42.5, 59.6)	51.2 (43.5, 60.7)	50.0 (41.7, 59.0)	0.112
eGFR (ml/min/1.732m^{2*})	76.1 (64.1, 89.4)	75.9 (65.8, 90.4)	76.2 (62.7, 88.1)	0.078
ALT (IU/L)	32.0 (21.0, 52.0)	32.0 (20.0, 52.0)	33.0 (21.0, 53.0)	0.287
AST (IU/L)	90.0 (44.0, 191.0)	87.0 (44.0, 187.0)	91.0 (43.0, 193.0)	0.703
Baseline cTnI (ng/mL)	1.0 (0.1, 5.5)	1.1 (0.1, 6.2)	0.9 (0.1, 5.1)	0.419
Peak cTnI (ng/mL)	9.6 (2.1, 27.2)	9.3 (2.0, 24.6)	9.9 (2.3, 29.3)	0.229
Baseline NT-proBNP (ng/mL)	301.8 (83.1, 1007.0)	252.5 (70.4, 749.2)	337.6 (94.4, 1203.0)	0.005
Peak NT-proBNP (ng/mL)	1186.0 (469.6, 2776.0)	1056.0 (429.4, 2186.0)	1289.0 (476.3, 3154.0)	< 0.001
hsCRP (mg/L)	5.5 (1.8, 11.0)	5.4 (1.8, 10.8)	5.6 (2.0, 11.2)	0.178
LDL-C (mmol/L)	2.6 (2.0, 3.2)	2.6 (2.0, 3.2)	2.6 (2.0, 3.2)	0.801
GRACE score	108.0 (89.0, 127.0)	107.0 (90.0, 125.0)	109.0 (88.0, 129.0)	0.413
Medication at discharge				
Aspirin	1142 (94.9)	484 (95.1)	658 (94.8)	0.206
Ticagrelor	565 (47.5)	251 (49.3)	314 (46.1)	0.300
Clopidogrel	607 (51.0)	255 (50.1)	352 (51.7)	0.628
ACEI/ARB	830 (69.0)	356 (69.9)	474 (68.3)	0.951
Beta Blocker	1008 (83.8)	443 (87.0)	565 (81.4)	0.065
Statins	1139 (94.7)	497 (97.6)	642 (92.5)	0.007
Adverse outcomes				
Death	84 (7.0)	24 (4.7)	60 (8.6)	0.011
reMI	71 (5.9)	27 (5.3)	44 (6.3)	0.529
reHF	22 (1.8)	12 (2.4)	10 (1.4)	0.340
Revascularization	195 (16.2)	71 (13.9)	124 (17.9)	0.081
Stroke	41 (3.4)	18 (3.5)	23 (3.3)	0.961
MACE	343 (28.5)	125 (24.6)	218 (31.4)	0.011

Continuous variables are presented as medians (25–75th percentiles), and categorical variables are reported as counts (%). ACEIs/ARBs indicates angiotensin-converting enzyme inhibitors/angiotensin receptor blockers; ALT, alanine aminotransferase; AST, aspartate aminotransferase; BMI, body mass index; CKD, chronic kidney disease; cTnI, cardiac troponin I; GRACE, the Global Registry of Acute Coronary Events; HF, heart failure; hsCRP, high-sensitivity C-reactive protein; LDL-C, low-density lipoprotein cholesterol; LVEF, left ventricular ejection fraction; MACE, major adverse cardiovascular event; MI, myocardial infarction; MVD, multiple vessels disease; reHF, rehospitalization caused by heart failure; reMI, recurrent myocardial infarction; STMEI, ST-segment elevation myocardial infarction; NT-proBNP, N-terminal pro B-type natriuretic peptide; STMEI, ST-segment elevation myocardial infarction; PAD, peripheral artery disease; PCI, percutaneous coronary intervention; TMAO, trimethylamine-N-oxide. *estimated glomerular filtration rate (eGFR) was calculated according to the Modification of Diet in Renal Disease formula.

Table S6. Association between choline levels at enrollment and all endpoints.

Endpoint	Group	Event (n.%)	Crude HR (95%CI)	*P*-value	Adjusted HR (95%CI) *	*P*-value
MACE						
	≤ median	155 (25.8)	[1]		[1]	
	> median	188 (31.2)	1.25 (1.01~1.55)	0.037	1.08 (0.86~1.35)	0.509
	Tertile 1	104 (25.9)	[1]		[1]	
	Tertile 2	116 (28.9)	1.13 (0.87~1.48)	0.350	1.12 (0.85~1.46)	0.425
	Tertile 3	123 (30.7)	1.20 (0.93~1.56)	0.167	1.02 (0.77~1.35)	0.878
	Trend. test	343 (28.5)	1.10 (0.96~1.25)	0.168	1.01 (0.88~1.16)	0.890
All-cause Death						
	≤ median	30 (5.0)	[1]		[1]	
	> median	54 (9.0)	1.86 (1.19~2.9)	0.007	1.08 (0.66~1.77)	0.764
	Tertile 1	16 (4.0)	[1]		[1]	
	Tertile 2	24 (6.0)	1.51 (0.80~2.84)	0.202	1.28 (0.67~2.45)	0.457
	Tertile 3	44 (11.0)	2.89 (1.63~5.13)	< 0.001	1.57 (0.84~2.94)	0.157
	Trend. test	84 (7.0)	1.74 (1.31~2.30)	< 0.001	1.25 (0.92~1.70)	0.153
reMI						
	≤ median	35 (5.8)	[1]		[1]	
	> median	36 (6.0)	1.05 (0.66~1.68)	0.827	0.94 (0.58~1.53)	0.803
	Tertile 1	27 (6.7)	[1]		[1]	
	Tertile 2	20 (5.0)	0.74 (0.41~1.31)	0.298	0.73 (0.41~1.33)	0.306
	Tertile 3	24 (6.0)	0.92 (0.53~1.59)	0.763	0.77 (0.43~1.38)	0.375
	Trend. test	71 (5.9)	0.95 (0.72~1.27)	0.746	0.87 (0.65~1.18)	0.375
reHF						
	≤ median	5 (0.8)	[1]		[1]	
	> median	17 (2.8)	3.56 (1.31~9.64)	0.013	2.90 (1.02~8.25)	0.045
	Tertile 1	4 (1.0)	[1]		[1]	
	Tertile 2	5 (1.2)	1.27 (0.34~4.72)	0.724	0.87 (0.21~3.57)	0.850
	Tertile 3	13 (3.2)	3.48 (1.13~10.67)	0.029	2.53 (0.78~8.28)	0.123
	Trend. test	22 (1.8)	2.00 (1.13~3.54)	0.017	1.76 (0.94~3.27)	0.076
Stroke						
	≤ median	16 (2.7)	[1]		[1]	
	> median	25 (4.2)	1.63 (0.87~3.06)	0.125	1.19 (0.61~2.31)	0.605
	Tertile 1	10 (2.5)	[1]		[1]	
	Tertile 2	13 (3.2)	1.32 (0.58~3.00)	0.515	1.03 (0.44~2.40)	0.950

	Tertile 3	18 (4.5)	1.94 (0.89~4.20)	0.094	1.40 (0.62~3.14)	0.416
	Trend. test	41 (3.4)	1.40 (0.95~2.06)	0.087	1.20 (0.80~1.80)	0.387
Revascularization						
	≤ median	101 (16.8)	[1]		[1]	
	> median	94 (15.6)	0.94 (0.71~1.24)	0.647	0.96 (0.72~1.29)	0.798
	Tertile 1	71 (17.7)	[1]		[1]	
	Tertile 2	73 (18.2)	1.04 (0.75~1.44)	0.830	1.08 (0.78~1.51)	0.641
	Tertile 3	51 (12.7)	0.72 (0.50~1.03)	0.069	0.77 (0.53~1.11)	0.164
	Trend. test	195 (16.2)	0.86 (0.72~1.02)	0.077	0.89 (0.74~1.06)	0.187

HR, hazard ratio; MACE, major adverse cardiovascular event; reHF, rehospitalization caused by heart failure; reMI, recurrent myocardial infarction. * Adjusted for the variables with $P < 0.1$ in the univariable models, including age, hypertension, diabetes, peripheral artery disease, chronic kidney disease, and previous history of stroke and MI, Killip II-IV, the Global Registry of Acute Coronary Events risk score, multiple vessels disease, percutaneous coronary intervention, and the peak value of cardiac troponin I and N-terminal pro B-type natriuretic peptide during hospitalization, as well as estimated glomerular filtration rate and left ventricular ejection fraction.

Table S7. Association between betaine levels at enrollment and all endpoints.

Endpoint	Group	Event (n.%)	Crude HR (95%CI)	*P*-value	Adjusted HR (95%CI) *	*P*-value
MACE						
	≤ median	175 (29.1)	[1]		[1]	
	> median	168 (27.9)	0.95 (0.77~1.17)	0.618	0.88 (0.71~1.11)	0.282
	Tertile 1	117 (29.2)	[1]		[1]	
	Tertile 2	111 (27.7)	0.93 (0.72~1.21)	0.603	0.87 (0.66~1.14)	0.307
	Tertile 3	115 (28.7)	0.97 (0.75~1.26)	0.819	0.92 (0.70~1.21)	0.552
	Trend. test	343 (28.5)	0.98 (0.86~1.12)	0.819	0.96 (0.83~1.10)	0.553
All-cause Death						
	≤ median	44 (7.3)	[1]		[1]	
	> median	40 (6.6)	0.91 (0.59~1.39)	0.658	0.84 (0.52~1.35)	0.466
	Tertile 1	27 (6.7)	[1]		[1]	
	Tertile 2	28 (7.0)	1.03 (0.61~1.75)	0.903	0.67 (0.37~1.21)	0.186
	Tertile 3	29 (7.2)	1.07 (0.63~1.81)	0.796	0.88 (0.50~1.56)	0.672
	Trend. test	84 (7.0)	1.04 (0.80~1.35)	0.796	0.94 (0.70~1.27)	0.686
reMI						
	≤ median	33 (5.5)	[1]		[1]	
	> median	38 (6.3)	1.16 (0.73~1.85)	0.539	0.90 (0.55~1.47)	0.683
	Tertile 1	19 (4.7)	[1]		[1]	
	Tertile 2	29 (7.2)	1.54 (0.86~2.74)	0.146	1.29 (0.72~2.33)	0.395
	Tertile 3	23 (5.7)	1.22 (0.66~2.24)	0.524	0.91 (0.49~1.72)	0.779
	Trend. test	71 (5.9)	1.09 (0.82~1.45)	0.547	0.95 (0.70~1.28)	0.724
reHF						
	≤ median	8 (1.3)	[1]		[1]	
	> median	14 (2.3)	1.74 (0.73~4.15)	0.212	1.78 (0.69~4.58)	0.230
	Tertile 1	6 (1.5)	[1]		[1]	
	Tertile 2	3 (0.7)	0.49 (0.12~1.97)	0.316	0.42 (0.10~1.84)	0.251
	Tertile 3	13 (3.2)	2.16 (0.82~5.69)	0.118	2.28 (0.76~6.85)	0.142
	Trend. test	22 (1.8)	1.65 (0.95~2.84)	0.073	1.79 (0.96~3.35)	0.066

Stroke						
	≤ median	18 (3.0)	[1]		[1]	
	> median	23 (3.8)	1.29 (0.70~2.39)	0.419	1.21 (0.62~2.32)	0.578
	Tertile 1	12 (3.0)	[1]		[1]	
	Tertile 2	13 (3.2)	1.09 (0.50~2.38)	0.833	1.14 (0.51~2.55)	0.742
	Tertile 3	16 (4.0)	1.35 (0.64~2.85)	0.434	1.30 (0.58~2.88)	0.524
	Trend. test	41 (3.4)	1.16 (0.80~1.70)	0.428	1.14 (0.76~1.70)	0.524
Revascularization						
	≤ median	100 (16.6)	[1]		[1]	
	> median	95 (15.8)	0.93 (0.70~1.23)	0.608	0.88 (0.66~1.19)	0.412
	Tertile 1	67 (16.7)	[1]		[1]	
	Tertile 2	65 (16.2)	0.96 (0.68~1.35)	0.829	0.91 (0.64~1.29)	0.595
	Tertile 3	63 (15.7)	0.92 (0.65~1.29)	0.619	0.90 (0.62~1.29)	0.554
	Trend. test	195 (16.2)	0.96 (0.81~1.14)	0.619	0.95 (0.79~1.14)	0.553

HR, hazard ratio; MACE, major adverse cardiovascular event; reHF, rehospitalization caused by heart failure; reMI, recurrent myocardial infarction. * Adjusted for the variables with $P < 0.1$ in the univariable models, including age, hypertension, diabetes, peripheral artery disease, chronic kidney disease, and previous history of stroke and MI, Killip II-IV, the Global Registry of Acute Coronary Events risk score, multiple vessels disease, percutaneous coronary intervention, and the peak value of cardiac troponin I and N-terminal pro B-type natriuretic peptide during hospitalization, as well as estimated glomerular filtration rate and left ventricular ejection fraction.

Table S8. Association between L-carnitine levels at visit 1 and all endpoints.

Endpoint	Group	Event (n.%)	Crude HR (95%CI)	*P*-value	Adjusted HR (95%CI) *	*P*-value
MACE						
	≤ median	166 (27.6)	[1]		[1]	
	> median	177 (29.4)	1.07 (0.87~1.33)	0.512	1.10 (0.89~1.38)	0.382
	Tertile 1	104 (25.9)	[1]		[1]	
	Tertile 2	112 (27.9)	1.09 (0.84~1.42)	0.524	1.12 (0.85~1.48)	0.405
	Tertile 3	127 (31.7)	1.25 (0.97~1.62)	0.088	1.26 (0.96~1.65)	0.090
	Trend. test	343 (28.5)	1.12 (0.98~1.28)	0.087	1.12 (0.98~1.29)	0.089
All-cause Death						
	≤ median	38 (6.3)	[1]		[1]	
	> median	46 (7.6)	1.21 (0.79~1.86)	0.389	1.29 (0.81~2.07)	0.279
	Tertile 1	24 (6.0)	[1]		[1]	
	Tertile 2	26 (6.5)	1.09 (0.62~1.89)	0.771	1.14 (0.62~2.07)	0.678
	Tertile 3	34 (8.5)	1.41 (0.84~2.38)	0.198	1.47 (0.83~2.60)	0.182
	Trend. test	84 (7.0)	1.19 (0.92~1.55)	0.189	1.22 (0.92~1.62)	0.177
reMI						
	≤ median	31 (5.2)	[1]		[1]	
	> median	40 (6.6)	1.30 (0.81~2.08)	0.273	1.20 (0.75~1.94)	0.447
	Tertile 1	21 (5.2)	[1]		[1]	
	Tertile 2	22 (5.5)	1.06 (0.58~1.93)	0.845	1.05 (0.57~1.92)	0.880
	Tertile 3	28 (7.0)	1.35 (0.76~2.37)	0.303	1.19 (0.67~2.12)	0.554

	Trend. test	71 (5.9)	1.17 (0.88~1.55)	0.295	1.09 (0.82~1.46)	0.547
reHF						
	≤ median	13 (2.2)	[1]		[1]	
	> median	9 (1.5)	0.69 (0.29~1.61)	0.387	0.77 (0.32~1.90)	0.574
	Tertile 1	8 (2.0)	[1]		[1]	
	Tertile 2	7 (1.7)	0.88 (0.32~2.41)	0.797	1.21 (0.41~3.58)	0.725
	Tertile 3	7 (1.7)	0.87 (0.32~2.41)	0.792	1.08 (0.36~3.23)	0.884
	Trend. test	22 (1.8)	0.93 (0.56~1.56)	0.790	1.04 (0.61~1.77)	0.886
Stroke						
	≤ median	20 (3.3)	[1]		[1]	
	> median	21 (3.5)	1.05 (0.57~1.94)	0.873	0.97 (0.52~1.84)	0.933
	Tertile 1	11 (2.7)	[1]		[1]	
	Tertile 2	14 (3.5)	1.29 (0.58~2.83)	0.533	1.29 (0.58~2.86)	0.539
	Tertile 3	16 (4.0)	1.46 (0.68~3.14)	0.336	1.30 (0.58~2.89)	0.520
	Trend. test	41 (3.4)	1.20 (0.82~1.75)	0.339	1.13 (0.77~1.68)	0.528
Revascularization						
	≤ median	93 (15.5)	[1]		[1]	
	> median	102 (16.9)	1.11 (0.84~1.47)	0.455	1.12 (0.84~1.50)	0.437
	Tertile 1	61 (15.2)	[1]		[1]	
	Tertile 2	61 (15.2)	1.01 (0.71~1.44)	0.970	0.99 (0.69~1.43)	0.977
	Tertile 3	73 (18.2)	1.22 (0.87~1.72)	0.247	1.24 (0.88~1.76)	0.225
	Trend. test	195 (16.2)	1.11 (0.93~1.32)	0.241	1.12 (0.94~1.33)	0.218

HR, hazard ratio; MACE, major adverse cardiovascular event; reHF, rehospitalization caused by heart failure; reMI, recurrent myocardial infarction. * Adjusted for the variables with $P < 0.1$ in the univariable models, including age, hypertension, diabetes, peripheral artery disease, chronic kidney disease, and previous history of stroke and MI, Killip II-IV, the Global Registry of Acute Coronary Events risk score, multiple vessels disease, percutaneous coronary intervention, and the peak value of cardiac troponin I and N-terminal pro B-type natriuretic peptide during hospitalization, as well as estimated glomerular filtration rate and left ventricular ejection fraction.

Table S9. Association between choline levels of V1 and V2 and all endpoints.

Endpoint	Group	Event (n.%)	Crude HR (95%CI)	*P*-value	Adjusted HR (95%CI) *	*P*-value	Adjusted HR (95%CI) #	*P*-value
MACE								
	L/L	27 (18.4)	[1]		[1]		[1]	
	L/H	24 (22.4)	1.23 (0.71~2.14)	0.454	1.31 (0.74~2.34)	0.358	1.33 (0.74~2.39)	0.340
	H/L	26 (24.3)	1.36 (0.79~2.33)	0.266	1.31 (0.74~2.31)	0.357	1.31 (0.74~2.31)	0.355
	H/H	42 (28.4)	1.71 (1.05~2.77)	0.030	1.69 (1.01~2.83)	0.047	1.70 (1.01~2.86)	0.045
	Trend. test	119 (23.4)	1.19 (1.02~1.39)	0.028	1.17 (1.00~1.38)	0.056	1.17 (1.00~1.38)	0.056
	Group 1	51 (20.1)	[1]		[1]		[1]	
	Group 2	26 (24.3)	1.24 (0.77~1.98)	0.378	1.16 (0.70~1.92)	0.559	1.16 (0.70~1.92)	0.565
	Group 3	42 (28.4)	1.55 (1.03~2.34)	0.034	1.50 (0.96~2.34)	0.073	1.50 (0.96~2.34)	0.072
	Trend. test	119 (23.4)	1.25 (1.02~1.53)	0.034	1.22 (0.98~1.53)	0.076	1.22 (0.98~1.53)	0.076
All-cause Death								
	L/L	5 (3.4)	[1]		[1]		[1]	
	L/H	6 (5.6)	1.72 (0.52~5.63)	0.373	1.59 (0.43~5.87)	0.490	1.81 (0.47~6.93)	0.385
	H/L	5 (4.7)	1.34 (0.39~4.63)	0.644	0.93 (0.23~3.76)	0.919	0.95 (0.24~3.84)	0.944
	H/H	8 (5.4)	1.71 (0.56~5.24)	0.345	0.87 (0.25~3.06)	0.830	0.92 (0.26~3.24)	0.899
	Trend. test	24 (4.7)	1.15 (0.81~1.61)	0.435	0.92 (0.62~1.36)	0.674	0.93 (0.63~1.37)	0.714
	Group 1	11 (4.3)	[1]		[1]		[1]	
	Group 2	5 (4.7)	1.04 (0.36~2.98)	0.949	0.75 (0.22~2.57)	0.648	0.73 (0.21~2.52)	0.623
	Group 3	8 (5.4)	1.32 (0.53~3.29)	0.547	0.71 (0.24~2.12)	0.544	0.72 (0.24~2.15)	0.558
	Trend. test	24 (4.7)	1.15 (0.72~1.81)	0.561	0.84 (0.49~1.45)	0.529	0.84 (0.49~1.46)	0.542
reMI								
	L/L	5 (3.4)	[1]		[1]		[1]	
	L/H	2 (1.9)	0.56 (0.11~2.90)	0.492	0.82 (0.15~4.34)	0.812	0.83 (0.15~4.50)	0.830
	H/L	8 (7.5)	2.22 (0.72~6.78)	0.163	2.97 (0.93~9.48)	0.066	2.96 (0.93~9.44)	0.067
	H/H	8 (5.4)	1.71 (0.56~5.25)	0.344	2.13 (0.65~6.96)	0.210	2.15 (0.66~7.03)	0.207
	Trend. test	23 (4.5)	1.29 (0.91~1.85)	0.155	1.37 (0.95~1.98)	0.091	1.38 (0.95~1.99)	0.089
	Group 1	7 (2.8)	[1]		[1]		[1]	
	Group 2	8 (7.5)	2.71 (0.98~7.47)	0.055	3.17 (1.11~9.01)	0.031	3.13 (1.09~8.97)	0.034
	Group 3	8 (5.4)	2.10 (0.76~5.78)	0.153	2.27 (0.77~6.66)	0.135	2.27 (0.78~6.67)	0.135
	Trend. test	23 (4.5)	1.43 (0.90~2.28)	0.129	1.52 (0.93~2.48)	0.098	1.52 (0.93~2.49)	0.098
reHF								
	L/L	0 (0)	[1]		[1]		[1]	
	L/H	2 (1.9)	74171112.77 (0~Inf)	0.997	152352873.18 (0~Inf)	0.998	121374977.96 (0~Inf)	0.997
	H/L	4 (3.7)	148948820.01 (0~Inf)	0.997	180257312.95 (0~Inf)	0.998	88712522.97 (0~Inf)	0.997
	H/H	5 (3.4)	137068286.14 (0~Inf)	0.997	131293570.64 (0~Inf)	0.998	78872116.51 (0~Inf)	0.997

	Trend. test	11 (2.2)	1.87 (1.03~3.42)	0.041	1.69 (0.60~4.77)	0.322	1.75 (0.61~5.05)	0.299
	Group 1	2 (0.8)	[1]		[1]		[1]	
	Group 2	4 (3.7)	4.78 (0.88~26.10)	0.071	3.21 (0.26~38.99)	0.360	2.81 (0.23~34.21)	0.417
	Group 3	5 (3.4)	4.40 (0.85~22.68)	0.077	2.70 (0.20~36.02)	0.453	2.68 (0.20~35.54)	0.454
	Trend. test	11 (2.2)	1.90 (0.95~3.81)	0.072	1.59 (0.47~5.35)	0.457	1.61 (0.47~5.53)	0.451
Stroke								
	L/L	2 (1.4)	[1]		[1]		[1]	
	L/H	5 (4.7)	3.46 (0.67~17.86)	0.137	3.18 (0.58~17.61)	0.185	3.41 (0.61~19.18)	0.163
	H/L	1 (0.9)	0.68 (0.06~7.54)	0.756	0.43 (0.03~5.68)	0.522	0.46 (0.04~6.07)	0.557
	H/H	10 (6.8)	5.26 (1.15~24.00)	0.032	4.69 (0.94~23.39)	0.059	5.26 (1.03~26.96)	0.046
	Trend. test	18 (3.5)	1.55 (1.01~2.37)	0.045	1.46 (0.91~2.35)	0.113	1.50 (0.93~2.41)	0.095
	Group 1	7 (2.8)	[1]		[1]		[1]	
	Group 2	1 (0.9)	0.34 (0.04~2.73)	0.308	0.21 (0.02~2.08)	0.182	0.22 (0.02~2.14)	0.191
	Group 3	10 (6.8)	2.59 (0.99~6.80)	0.054	2.38 (0.82~6.92)	0.111	2.54 (0.86~7.51)	0.091
	Trend. test	18 (3.5)	1.67 (0.98~2.85)	0.059	1.55 (0.86~2.81)	0.144	1.60 (0.88~2.91)	0.124
Revascularization								
	L/L	21 (14.3)	[1]		[1]		[1]	
	L/H	13 (12.1)	0.85 (0.42~1.69)	0.637	0.94 (0.46~1.94)	0.866	0.92 (0.44~1.92)	0.818
	H/L	15 (14.0)	0.98 (0.05~1.90)	0.951	1.13 (0.58~2.23)	0.716	1.13 (0.58~2.24)	0.715
	H/H	20 (13.5)	0.97 (0.53~1.79)	0.926	1.22 (0.63~2.35)	0.562	1.20 (0.62~2.33)	0.595
	Trend. test	69 (13.6)	1.00 (0.82~1.22)	0.991	1.08 (0.87~1.33)	0.497	1.07 (0.87~1.33)	0.510
	Group 1	34 (13.4)	[1]		[1]		[1]	
	Group 2	15 (14.0)	1.05 (0.57~1.92)	0.882	1.16 (0.62~2.16)	0.637	1.17 (0.63~2.19)	0.619
	Group 3	20 (13.5)	1.04 (0.60~1.81)	0.892	1.25 (0.68~2.27)	0.473	1.24 (0.68~2.26)	0.483
	Trend. test	69 (13.6)	1.02 (0.78~1.34)	0.882	1.12 (0.83~1.50)	0.449	1.12 (0.83~1.50)	0.454

Patients were divided into four groups according to choline levels at the enrollment (V1) and follow-up visit (V2) relative to the median of each visit point (1.2 μmol/L and 1.7 μmol/L for V1 and V2, respectively). L/L, low V1 and low V2; L/H, low V1 and high V2; H/L, high V1 and low V2; H/H, high V1 and high V2. Group1: patients with choline levels below the median at V1; Group 2: patients with higher levels of choline at V1 and subsequently lower levels at V2; Group3: patients with both higher choline levels at V1 and V2. HR, hazard ratio; MACE, major adverse cardiovascular event; reHF, rehospitalization caused by heart failure; reMI, recurrent myocardial infarction; TMAO, trimethylamine-N-oxide. * Model 1: Adjusted for the variables with $P < 0.1$ in the univariable models, including age, hypertension, diabetes, peripheral artery disease, chronic kidney disease, and previous history of stroke and MI, Killip II-IV, the Global Registry of Acute Coronary Events risk score, multiple vessels disease, percutaneous coronary intervention, and the peak value of cardiac troponin I and N-terminal pro B-type natriuretic peptide during hospitalization, as well as estimated glomerular filtration rate and left ventricular ejection fraction at V2. # Model 2: Model 1+ TMAO levels at V2.

Table S10. Association between betaine levels of V1 and V2 and all endpoints.

Endpoint	Group	Event (n.%)	Crude HR (95%CI)	*P*-value	Adjusted HR (95%CI) *	*P*-value
MACE						
	L/L	37 (25.9)	[1]		[1]	

	L/H	24 (21.6)	0.84 (0.50~1.41)	0.514	0.88 (0.51~1.51)	0.634
	H/L	25 (22.5)	0.87 (0.52~1.44)	0.584	0.81 (0.47~1.41)	0.453
	H/H	33 (22.9)	0.91 (0.57~1.46)	0.695	0.87 (0.53~1.43)	0.583
	Trend. test	119 (23.4)	0.97 (0.83~1.13)	0.718	0.95 (0.81~1.12)	0.551
All-cause Death						
	L/L	9 (6.3)	[1]		[1]	
	L/H	5 (4.5)	0.76 (0.25~2.27)	0.620	0.76 (0.19~2.98)	0.692
	H/L	6 (5.4)	0.88 (0.31~2.47)	0.807	0.66 (0.19~2.28)	0.513
	H/H	4 (2.8)	0.46 (0.14~1.50)	0.199	0.38 (0.10~1.40)	0.145
	Trend. test	24 (4.7)	0.82 (0.58~1.15)	0.249	0.74 (0.50~1.11)	0.144
reMI						
	L/L	7 (4.9)	[1]		[1]	
	L/H	3 (2.7)	0.58 (0.15~2.25)	0.432	0.62 (0.15~2.50)	0.497
	H/L	6 (5.4)	1.13 (0.38~3.37)	0.825	1.10 (0.35~3.43)	0.866
	H/H	7 (4.9)	1.04 (0.37~2.98)	0.935	0.95 (0.32~2.83)	0.932
	Trend. test	23 (4.5)	1.06 (0.75~1.50)	0.741	1.03 (0.72~1.46)	0.891
reHF						
	L/L	3 (2.1)	[1]		[1]	
	L/H	1 (0.9)	0.43 (0.04~4.12)	0.463	0.38 (0.02~8.90)	0.547
	H/L	3 (2.7)	1.29 (0.26~6.37)	0.758	2.67 (0.21~34.21)	0.450
	H/H	4 (2.8)	1.33 (0.30~5.93)	0.710	1.13 (0.12~10.38)	0.915
	Trend. test	11 (2.2)	1.18 (0.71~1.97)	0.525	1.13 (0.57~2.27)	0.722
Stroke						
	L/L	4 (2.8)	[1]		[1]	
	L/H	3 (2.7)	0.97 (0.22~4.31)	0.963	1.10 (0.23~5.35)	0.903
	H/L	6 (5.4)	1.93 (0.55~6.85)	0.307	1.85 (0.46~7.49)	0.388
	H/H	5 (3.5)	1.25 (0.34~4.66)	0.738	1.12 (0.27~4.55)	0.875
	Trend. test	18 (3.5)	1.13 (0.76~1.68)	0.546	1.07 (0.69~1.63)	0.771
Revascularization						
	L/L	19 (13.3)	[1]		[1]	
	L/H	17 (15.3)	1.19 (0.62~2.29)	0.604	1.21 (0.61~2.38)	0.583
	H/L	15 (13.5)	1.02 (0.52~2.00)	0.959	0.87 (0.43~1.79)	0.714
	H/H	18 (12.5)	0.95 (0.50~1.81)	0.871	0.84 (0.43~1.64)	0.606
	Trend. test	69 (13.6)	0.97 (0.80~1.19)	0.781	0.93 (0.75~1.14)	0.479

Patients were divided into four groups according to betaine levels at the enrollment (V1) and follow-up visit (V2) relative to the median of each visit point (1.9 μmol/L and 3.1μmol/L for V1 and V2, respectively). L/L, low V1 and low V2; L/H, low V1 and high V2; H/L, high V1 and low V2; H/H, high V1 and high V2. HR, hazard ratio; MACE, major adverse cardiovascualr event; reHF, rehospitalization caused by heart failure; reMI, recurrent myocardial infarction. * Adjusted for the variables with $P < 0.1$ in the univariable models, including age, hypertension, diabetes, peripheral artery disease, chronic kidney disease, and previous history of stroke and MI, Killip II-IV, the Global Registry of Acute Coronary Events risk score, multiple vessels disease, percutaneous coronary intervention, and the peak value of cardiac troponin I and N-terminal pro B-type natriuretic peptide during hospitalization, as well as estimated glomerular filtration rate and left ventricular ejection fraction at V2.

Table S11. Association between L-carnitine levels of V1 and V2 and all endpoints.

Endpoint	Group	Event (n.%)	Crude HR (95%CI)	*P*-value	Adjusted HR (95%CI) *	*P*-value
MACE						
	L/L	37 (24.0)	[1]		[1]	
	L/H	25 (25.0)	1.07 (0.65~1.78)	0.788	1.02 (0.59~1.76)	0.952
	H/L	19 (19.0)	0.77 (0.44~1.34)	0.358	0.70 (0.39~1.26)	0.234
	H/H	38 (24.5)	1.06 (0.67~1.67)	0.804	1.06 (0.66~1.72)	0.801

	Trend. test	119 (23.4)	1.00 (0.86~1.16)	0.970	1.00 (0.85~1.17)	0.984
All-cause Death						
	L/L	5 (3.2)	[1]		[1]	
	L/H	7 (7.0)	2.30 (0.73~7.24)	0.156	1.77 (0.44~7.07)	0.418
	H/L	6 (6.0)	1.83 (0.56~5.98)	0.320	1.28 (0.30~5.34)	0.739
	H/H	6 (3.9)	1.24 (0.38~4.06)	0.724	1.31 (0.35~4.87)	0.692
	Trend. test	24 (4.7)	1.03 (0.74~1.44)	0.844	1.03 (0.70~1.53)	0.866
reMI						
	L/L	8 (5.2)	[1]		[1]	
	L/H	1 (1.0)	0.20 (0.03~1.60)	0.130	0.19 (0.02~1.56)	0.122
	H/L	6 (6.0)	1.16 (0.40~3.33)	0.787	1.13 (0.36~3.56)	0.832
	H/H	8 (5.2)	1.04 (0.39~2.77)	0.941	0.99 (0.34~2.90)	0.992
	Trend. test	23 (4.5)	1.09 (0.77~1.53)	0.630	1.08 (0.74~1.55)	0.698
reHF						
	L/L	3 (1.9)	[1]		[1]	
	L/H	4 (4.0)	2.12 (0.47~9.48)	0.325	36.05 (1.04~1253.5)	0.048
	H/L	2 (2.0)	1.03 (0.17~6.18)	0.972	4.12 (0.16~106.26)	0.393
	H/H	2 (1.3)	0.66 (0.11~3.96)	0.652	5.23 (0.18~149.17)	0.333
	Trend. test	11 (2.2)	0.85 (0.52~1.40)	0.526	1.12 (0.56~2.27)	0.747
Stroke						
	L/L	6 (3.9)	[1]		[1]	
	L/H	2 (2.0)	0.52 (0.11~2.60)	0.430	0.55 (0.08~3.52)	0.524
	H/L	1 (1.0)	0.25 (0.03~2.12)	0.206	0.30 (0.03~2.78)	0.291
	H/H	9 (5.8)	1.54 (0.55~4.32)	0.415	1.72 (0.53~5.61)	0.370
	Trend. test	18 (3.5)	1.17 (0.80~1.73)	0.421	1.24 (0.82~1.88)	0.316
Revascularization						
	L/L	24 (15.6)	[1]		[1]	
	L/H	12 (12.0)	0.78 (0.39~1.56)	0.486	0.62 (0.29~1.31)	0.210
	H/L	11 (11.0)	0.70 (0.34~1.42)	0.322	0.66 (0.32~1.38)	0.273
	H/H	22 (14.2)	0.93 (0.52~1.66)	0.815	0.86 (0.47~1.58)	0.632
	Trend. test	69 (13.6)	0.97 (0.80~1.18)	0.756	0.96 (0.78~1.18)	0.670

Patients were divided into four groups according to L-carnitine levels at the enrollment (V1) and follow-up visit (V2) relative to the median of each visit point (51.2 μmol/L and 67.0μmol/L for V1 and V2, respectively). L/L, low V1 and low V2; L/H, low V1 and high V2; H/L, high V1 and low V2; H/H, high V1 and high V2. HR, hazard ratio; MACE, major adverse cardiovascular event; reHF, rehospitalization caused by heart failure; reMI, recurrent myocardial infarction. * Adjusted for the variables with $P < 0.1$ in the univariable models, including age, hypertension, diabetes, peripheral artery disease, chronic kidney disease, and previous history of stroke and MI, Killip II-IV, the Global Registry of Acute Coronary Events risk score, multiple vessels disease, percutaneous coronary intervention, and the peak value of cardiac troponin I and N-terminal pro B-type natriuretic peptide during hospitalization, as well as estimated glomerular filtration rate and left ventricular ejection fraction at V2.

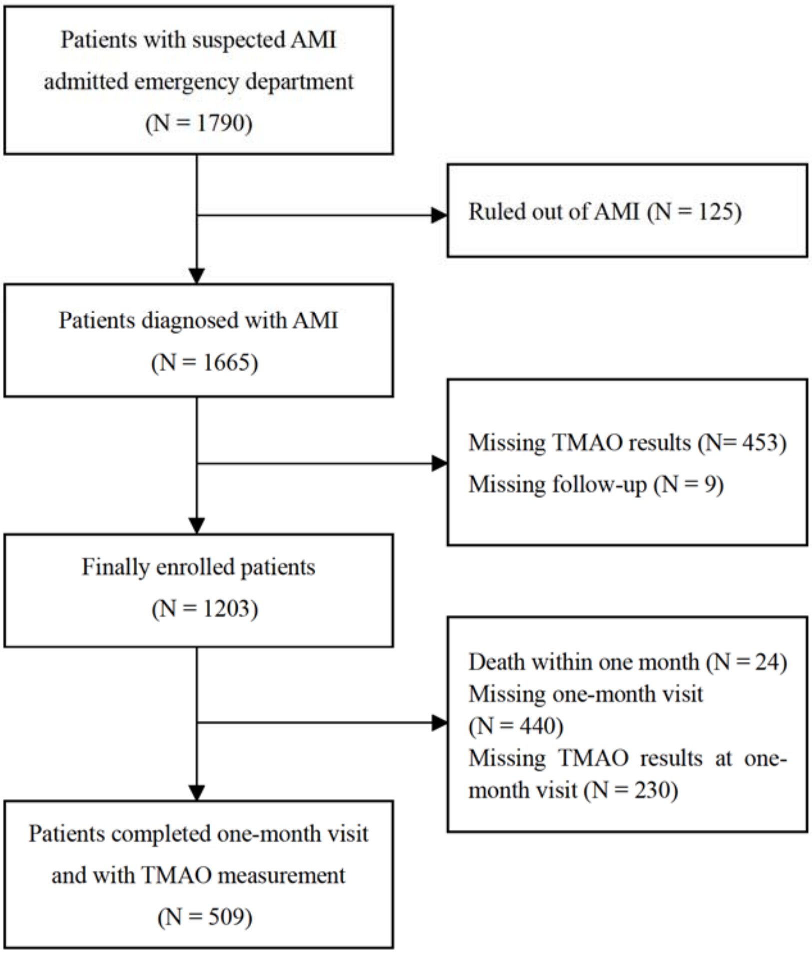

Figure S1. The flowchart of the patient selection process. AMI, acute myocardial infarction; TMAO, trimethylamine-N-oxide.

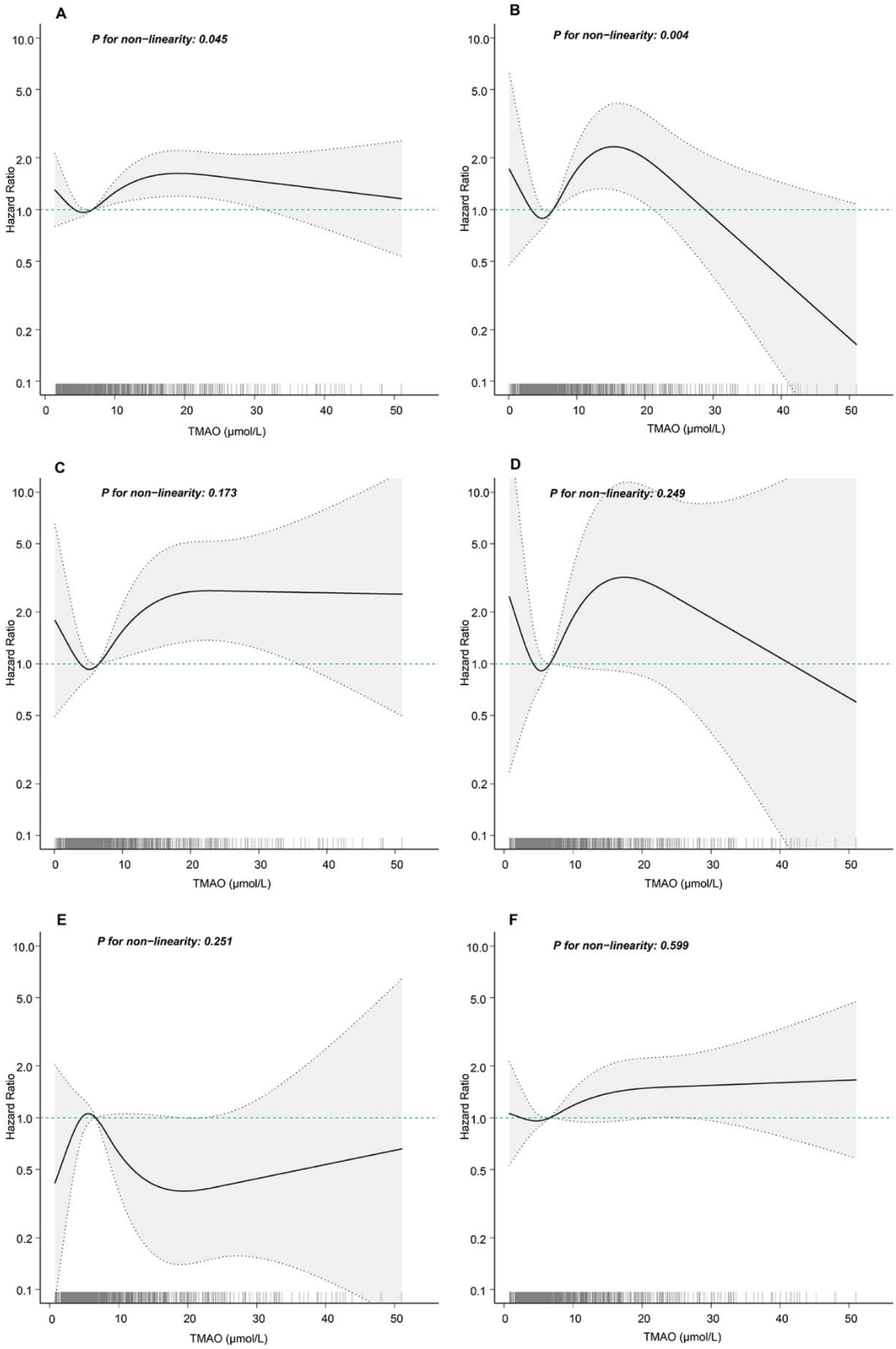

Figure S2. Continuous hazard ratios across TMAO levels at enrollment for major adverse cardiovascular event (**A**), all-cause death (**B**), myocardial infarction (**C**), rehospitalization caused by heart failure (**D**), stroke (**E**), and revascularization (**F**). TMAO, trimethylamine-N-oxide.

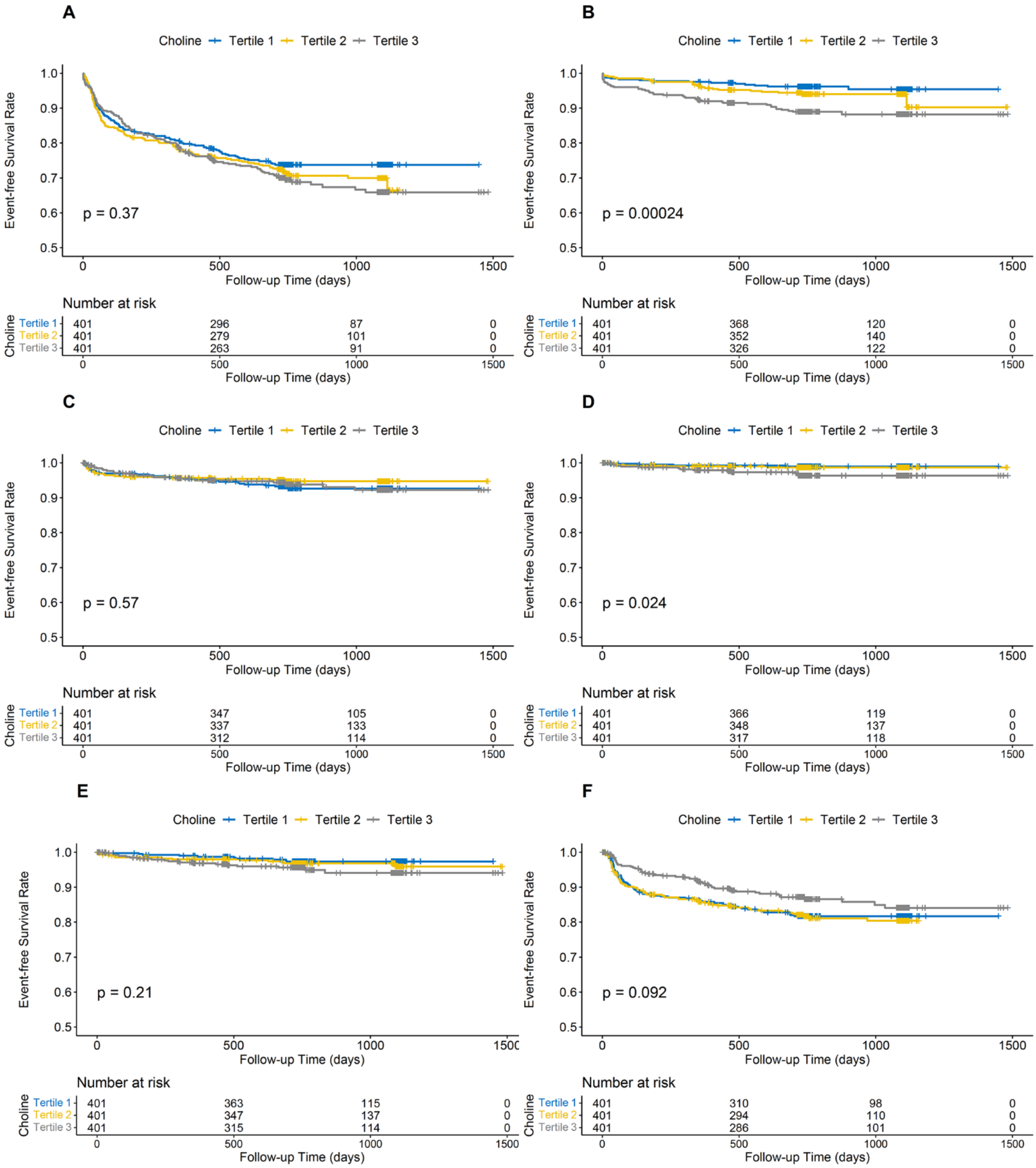

Figure S3. Kaplan-Meier curve for cumulative event-free survival in groups stratified by choline tertile levels at enrollment. **A**: major adverse cardiovascular event; **B**: all-cause death; **C**: myocardial infarction; **D**: rehospitalization caused by heart failure; **E**: stroke; **F**: revascularization.

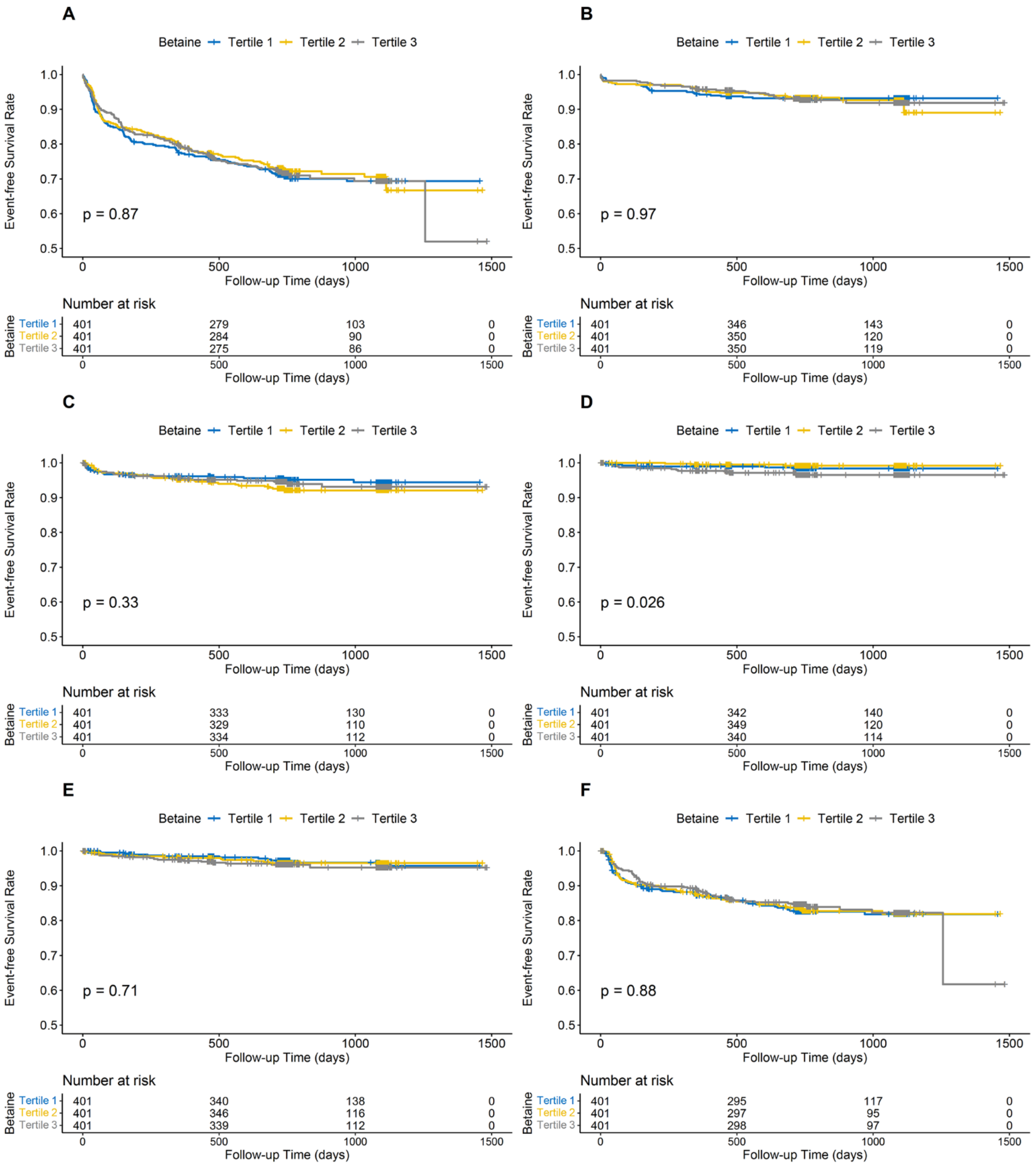

Figure S4. Kaplan-Meier curve for cumulative event-free survival in groups stratified by betaine tertile levels at enrollment. **A**: major adverse cardiovascular event; **B**: all-cause death; **C**: myocardial infarction; **D**: rehospitalization caused by heart failure; **E**: stroke; **F**: revascularization.

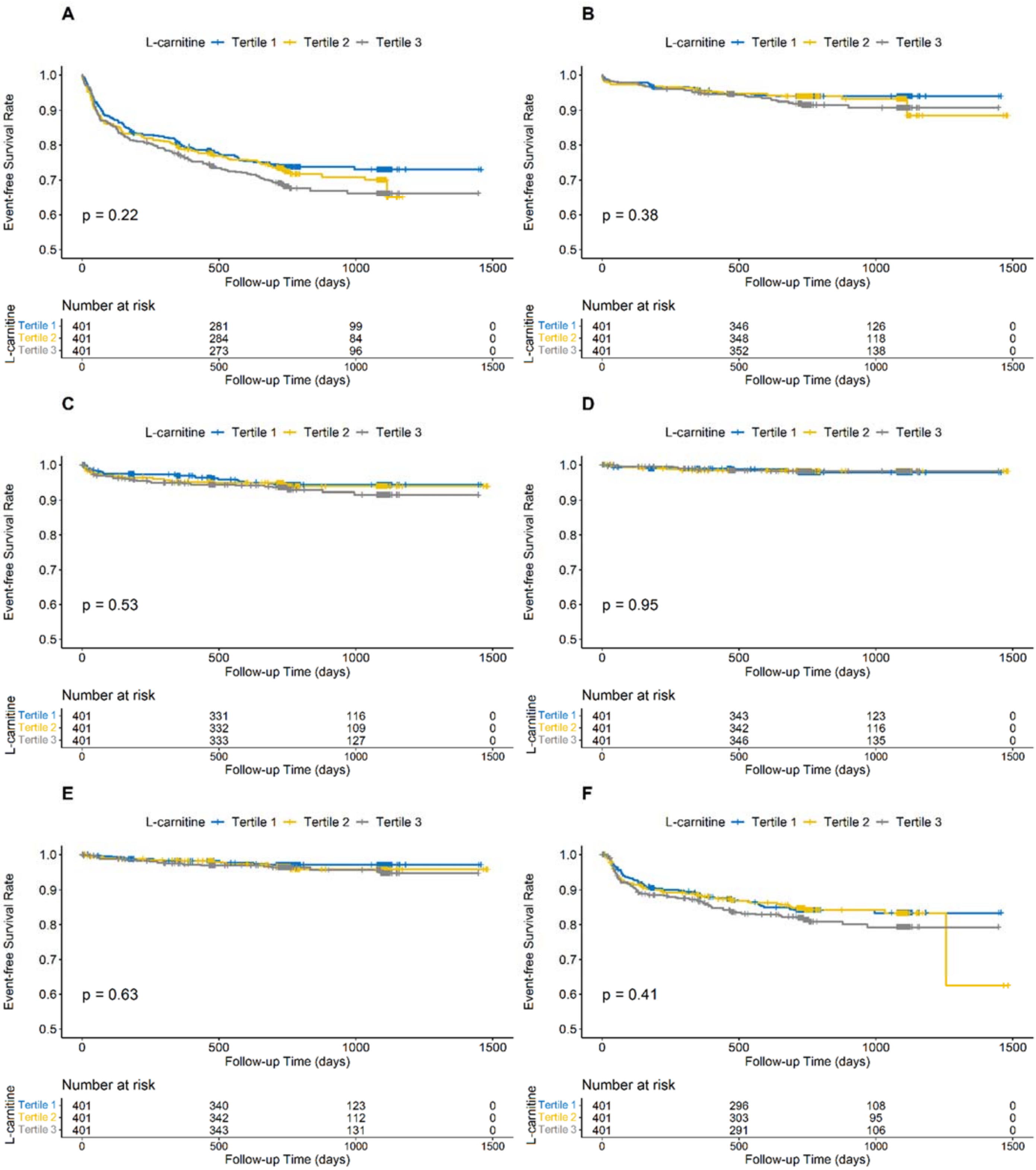

Figure S5. Kaplan-Meier curve for cumulative event-free survival in groups stratified by L-carnitine tertile levels at enrollment. **A**: major adverse cardiovascular event; **B**: all-cause death; **C**: myocardial infarction; **D**: rehospitalization caused by heart failure; **E**: stroke; **F**: revascularization.

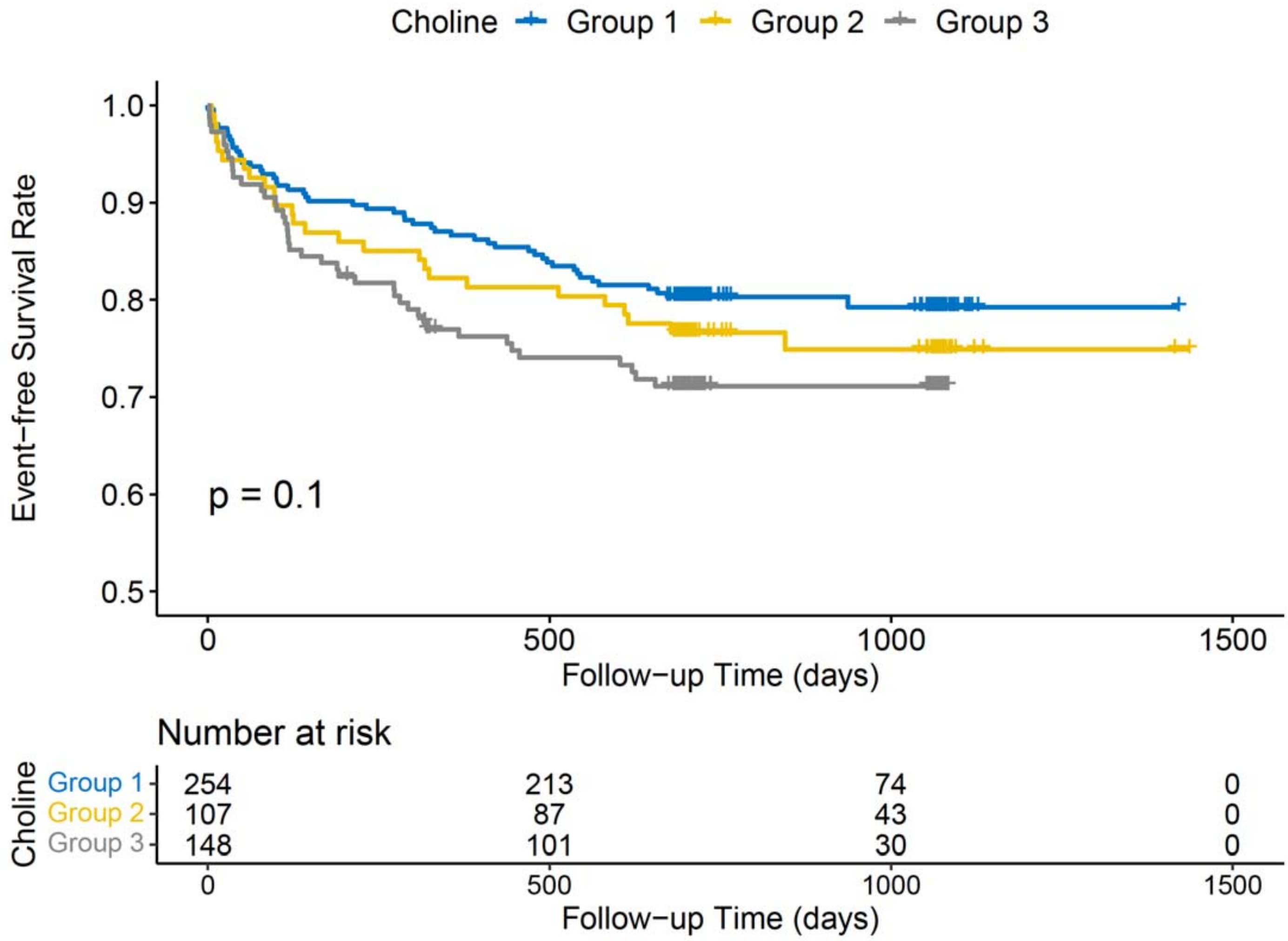

Figure S6. Kaplan-Meier curve for cumulative MACE-free survival in groups stratified by choline levels at enrollment (V1) and follow-up visit (V2). Patients were divided into three groups according to the respective median levels of choline at V1 and V2, namely Group 1 (patients with low choline levels at V1), Group 2 (patients with high choline levels at V1 and subsequently low levels at V2) and Group 3 (patients with high choline levels at V1 and V2). MACE, major adverse cardiovascular event.
